# Single-cell and spatial proteo-transcriptomic profiling reveals immune infiltration heterogeneity associated with neuroendocrine features in small cell lung cancer

**DOI:** 10.1038/s41421-024-00703-x

**Published:** 2024-09-04

**Authors:** Ying Jin, Yuefeng Wu, Alexandre Reuben, Liang Zhu, Carl M. Gay, Qingzhe Wu, Xintong Zhou, Haomin Mo, Qi Zheng, Junyu Ren, Zhaoyuan Fang, Teng Peng, Nan Wang, Liang Ma, Lungevity PANSHI Initiative Consortium, Qingzhe Wu, Nan Wang, Yuefeng Wu, Hai Song, Yun Fan, Hai Song, Jianjun Zhang, Ming Chen

**Affiliations:** 1 Zhejiang Cancer Hospital, Hangzhou Institute of Medicine (HIM), Chinese Academy of Sciences, Hangzhou, Zhejiang China; 2 Zhejiang Key Laboratory of Radiation Oncology, Hangzhou, Zhejiang China; 3 The MOE Key Laboratory of Biosystems Homeostasis and Protection, Zhejiang Provincial Key Laboratory for Cancer Molecular Cell Biology and Innovation Center for Cell Signaling Network, Life Sciences Institute, Zhejiang University, Hangzhou, Zhejiang China; 4 School of Life Sciences, Westlake University, Hangzhou, Zhejiang China; 5 Department of Cardiovascular Surgery, The First Affiliated Hospital, School of Medicine, Zhejiang University, Hangzhou, Zhejiang China; 6 Zhejiang University-University of Edinburgh Institute (ZJU-UoE), School of Medicine, Zhejiang University, Haining, Zhejiang China; 7 Department of Thoracic/Head and Neck Medical Oncology, Division of Cancer Medicine, The University of Texas MD Anderson Cancer Center, Houston, TX USA; 8 College of Information Science and Electronic Engineering, Zhejiang University, Hangzhou, Zhejiang China; 9 Cosmos Wisdom Biotech Co. Ltd., Hangzhou, Zhejiang China; 10 Center for Oncology Medicine, The Fourth Affiliated Hospital of School of Medicine, and International School of Medicine, International Institutes of Medicine, Zhejiang University, Yiwu, Zhejiang China; 11 Department of Genomic Medicine, Division of Cancer Medicine, The University of Texas MD Anderson Cancer Center, Houston, TX USA; 12 State Key Laboratory of Oncology in South China, Guangdong Key Laboratory of Nasopharyngeal Carcinoma Diagnosis and Therapy, Guangdong Provincial Clinical Research Center for Cancer, Sun Yat-sen University Cancer Center, Guangzhou, Guangdong China; 13 United Laboratory of Frontier Radiotherapy Technology of Sun Yat-sen University & Chinese Academy of Sciences Ion Medical Technology Co., Ltd, Guangzhou, Guangdong China; 14 Westlake Laboratory of Life Sciences and Biomedicine, School of Life Sciences, Westlake University, Hangzhou, Zhejiang China

**Keywords:** Cancer microenvironment, Small-cell lung cancer

## Abstract

Small cell lung cancer (SCLC) is an aggressive pulmonary neuroendocrine malignancy featured by cold tumor immune microenvironment (TIME), limited benefit from immunotherapy, and poor survival. The spatial heterogeneity of TIME significantly associated with anti-tumor immunity has not been systemically studied in SCLC. We performed ultra-high-plex Digital Spatial Profiling on 132 tissue microarray cores from 44 treatment-naive limited-stage SCLC tumors. Incorporating single-cell RNA-sequencing data from a local cohort and published SCLC data, we established a spatial proteo-transcriptomic landscape covering over 18,000 genes and 60 key immuno-oncology proteins that participate in signaling pathways affecting tumorigenesis, immune regulation, and cancer metabolism across 3 pathologically defined spatial compartments (pan-CK-positive tumor nest; CD45/CD3-positive tumor stroma; para-tumor). Our study depicted the spatial transcriptomic and proteomic TIME architecture of SCLC, indicating clear intra-tumor heterogeneity dictated via canonical neuroendocrine subtyping markers; revealed the enrichment of innate immune cells and functionally impaired B cells in tumor nest and suggested potentially important immunoregulatory roles of monocytes/macrophages. We identified RE1 silencing factor (REST) as a potential biomarker for SCLC associated with low neuroendocrine features, more active anti-tumor immunity, and prolonged survival.

## Introduction

Small cell lung cancer (SCLC) accounts for ~15% of all histological lung cancer subtypes and is very aggressive, with extremely poor survival^1,2^. Pathophysiologically, SCLC is a neuroendocrine (NE) malignancy that is characterized by positive for chromogranin A (CgA), Synapsin I (Syn), and neural cell adhesion molecule 1 (NCAM1, also named CD56) based on immunohistochemical analysis^3^. Patients with limited-stage SCLC (LS-SCLC) are treated by concurrent chemotherapy and thoracic radiotherapy^4^. Treatment for patients with extensive disease (ES-SCLC) includes systemic chemotherapy (cisplatin or carboplatin plus etoposide) combined with immune checkpoint inhibitors (ICIs) targeting the programmed cell death ligand 1 (PD-L1)/programmed cell death protein 1 (PD-1) pathway. However, the clinical benefit from ICI is limited for SCLC patients compared with other cancer types^5^ despite that most SCLC patients are smokers and that the tumors usually have high tumor mutational burden (TMB), both of which have been reported to be associated with clinical benefit for ICI^6,7^.

PD-L1 expression, a biomarker to predict ICI responses across various cancers^8^, is extremely low in SCLC, suggestive of an uninflamed tumor immune microenvironment (TIME)^9–11^. Moreover, the TIME of SCLC is also heterogeneous^12^, which may further dampen the anti-tumor immune response^13^. Immune infiltration in both tumor nest and stroma has been reported to be associated with cellular plasticity driven by NE differentiation^14^ as well as other unknown intra-/interpatient heterogeneous factors within the TIME.

A tumor-centric classification has been proposed at the transcriptional level, with four SCLC subtypes based on their NE status. SCLC-A is characterized by high achaete-scute homolog 1 (ASCL1) expression; SCLC-N is defined by neurogenic differentiation factor 1 (NEUROD1) expression; SCLC-P is characterized by upregulated POU domain, class 2, transcription factor 3 (POU2F3) expression; and SCLC-Y is characterized by expression of the Yes-associated protein 1 (YAP1)^15^. SCLC-A and SCLC-N are believed to be neuroendocrine-high (NE-high) compared to SCLC-Y and SCLC-P subtypes, which are deemed as NE-low. NE-low subtypes may respond better to ICI therapy^14,16^, reportedly due to superior major histocompatibility complex (MHC) I antigen presentation and functionally competent CD8^+^ T cells^14^. The TIME of NE-high and NE-low SCLC has been previously defined using immunohistochemistry (IHC), suggesting probable microenvironment-directed patient stratification for ICI administration^17^. A more recent study identified the SCLC-I (I for inflammation) subtype that is associated with a higher level of immune infiltration and superior benefit from ICI^18^.

Dynamic tumor–stroma interactions play an important role in oncogenesis and TIME modulation^19^. The stroma mainly consists of the basement membrane, fibroblasts, extracellular matrix, and vasculature, providing essential nutrients and support for an array of immune cells. The dynamics of immune cell migration and cellular crosstalk across tumor nest and stroma are believed to eminently impact therapeutic efficiencies. Qualitative IHC has demonstrated that immune cells are mainly localized in the stroma, with very few infiltrating the tumor nest^17^. However, the broad spectrum of spatial interplay in SCLC has yet to be characterized^20^.

Tumor microenvironment-driven research in SCLC remains sparse^21^. In particular, the immune subsets and their abundance, the interaction between tumor nest and stroma, and the migration of immune cells in SCLC have not been systemically studied, largely due to the scarcity of resected SCLC tumors and the lack of a sub-histologically definable sampling strategy to generate high-plex multi-omics profiles across tumor and stromal compartments. To fill this void, we retrospectively and prospectively collected SCLC specimens and applied single-cell RNA-sequencing (scRNA-seq) and digital spatial profiling (DSP) to study the interactions between tumors and immune cells in the context of NE signals. We first obtained scRNA-seq data of 111,072 cells from 19 samples (primary tumors, metastases, and peripheral blood mononuclear cells (PBMCs)) from three patients with SCLC and two patients with large cell NE carcinoma (LCNEC) as control using 10x Chrominium technology. Meanwhile, formalin-fixed paraffin-embedded (FFPE) specimens of 16 SCLC tumors and 4 para-tumor lung tissues were subjected to 10x Flex scRNA-seq resulting 189,717 cells. We established a broad region-directed spatial proteo-transcriptomic (SPT) landscape of tumor nest, tumor stroma, and distant lung tissue from 44 treatment-naive SCLC patients using DSP^22^ covering 18,000 transcripts and 60 key immune modulatory proteins. We delineated the intra-tumor heterogeneity (ITH) and the relationship between tumor and stroma, which identified a RE1 silencing factor (REST)-mediated regulatory axis in a subset of patients that may have superior benefit from ICI therapy. We further explored publicly available SCLC bulk and scRNA-seq datasets to validate our spatial and communication analyses.

## Results

### scRNA profiling delineates NE features associated with immune infiltration and cell–cell interaction (CCI)

**Fig. 1:**
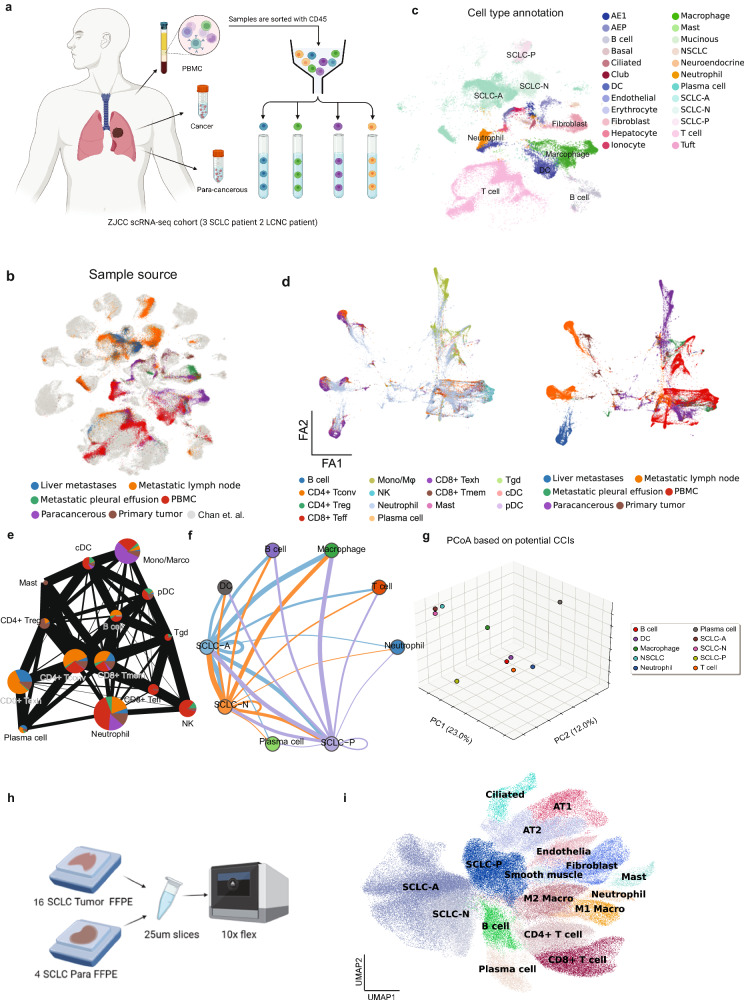
NE features and tumor spatial locations were associated with immune cell abundance and interactions. **a** Schematic diagram of the ZJCC scRNA-seq study design. **b** Projection of the data onto the Chan et al. reference dataset for precise cell type classification on Uniform Manifold Approximation and Projection (UMAP). Different colors represent sample sources. **c** Annotations of SCLC cells of different molecular subtypes (SCLC-A, SCLC-N, SCLC-P) and non-malignant cells. **d** Standard PAGA graph representing the topological information of the main immune cell types and spatial locations. **e** The network of main immune subsets from different tissues. Colors of dots in the network represent different sources. The size of the dots represents the abundance of each cell type. The weight of the lines represents the closeness of the interaction between each pair of immune subsets. **f** Crosstalk between cancer cells and immune cells via receptors and ligands identified by CellPhoneDB. Line weight represents the probability of cellular crosstalk. **g** Cell types projected with PCoA into Euclidean distance based on their potential CCI interaction. **h** Schematic of the 10x Flex FFPE sample scRNA-seq study design. **i** UMAP showing projection of the 10x Flex data with cell type classification.

Given the potential influence of NE characteristics on TIME in SCLC patients^21^, our investigation began with an examination of the correlation between NE features and SCLC TIME through scRNA-seq. We analyzed nineteen samples obtained from three SCLC patients and two LCNEC patients, encompassing primary tumors (*n* = 3), liver metastases (*n* = 2), lymph node metastases (*n* = 4), malignant pleural effusions (*n* = 2), tumor-adjacent normal lung tissues (*n* = 4), and matched PBMCs from the same patients (*n* = 5) (Fig. 1a). To enhance statistical power, we integrated our dataset with publicly available scRNA-seq data from Chan et al.^23^. In total, 111,072 cells with scRNA-seq transcriptomic profiles underwent subsequent analysis. Notably, a consistent cellular clustering pattern was observed between internal and external scRNA-seq data, primarily distinguished by tissue-of-origin (Fig. 1b). The analysis revealed a diverse TIME composition comprising 24 major cell types (Fig. 1c). Notably, tumor epithelium representing three molecular SCLC subtypes (SCLC-A, SCLC-N, and SCLC-P), characterized by distinct expression of NE biomarkers, was identified (Fig. 1c). Furthermore, developmental trajectory analysis using DDRtree (an algorithm integrated into pseudo-time analysis) confirmed the association of two marker genes (*ASCL1* and *POU2F3*) with distinct NE status, with NE-high (*ASCL1*-high) and NE-low cells (*POU2F3*-high) appearing in different branches (Supplementary Fig. S1a, b).

We subsequently reorganized the data, categorizing it based on either tissue-of-origin phenotypes or major functional immune cell types (Fig. 1d). PBMCs exhibited a diverse array of immune subsets (Fig. 1d, e) with CD4^+^/CD8^+^ T effector/memory (Teff/Tmem) cells and monocytes/dendritic cells (Monos/DCs) representing the majority, while CD8^+^ exhausted T cells (Texh) and CD4^+^ regulatory T cells (Treg) were less common (Fig. 1d, right). Regarding immune cell composition, primary and metastatic tumors were mainly characterized by CD8^+^ Texh, CD4^+^ Treg, neutrophils, and CD4^+^ conventional T cells (Tconv), whereas the para-tumor region was dominated by granulocyte lineages, including neutrophils, monocytes, and macrophages (Supplementary Fig. S1a).

To delve deeper into the interaction between NE-high (SCLC-A and SCLC-N) and NE-low (SCLC-P) cancer cells and immune cells, we computed CCI scores between cancer cells and seven key immune cell subsets (Fig. 1f). While different tumor subtypes (SCLC-A, -N, -P) displayed close interactions among themselves, they exhibited varying levels of epithelium–immune crosstalk with B cells, T cells, neutrophils, DCs, and plasma cells (Fig. 1f). Particularly noteworthy was the strong association of all subtypes with macrophages (Fig. 1f), a trend further supported by individual crosstalk component analysis via principal component analysis (PCA) (Fig. 1g), wherein SCLC-A and SCLC-N showed close relation, while macrophages demonstrated substantial independence from other immune cell types, suggesting a potentially unique role within the TIME (Fig. 1f).

Thus far, our preliminary data have suggested the presence of heterogeneous neoplastic cell populations within patients, along with shared and distinct tumor–immune interactions. However, these findings have been constrained by the limited number of patients analyzed. To address this, we extended our analysis to include an independent cohort comprising 16 SCLC tumors and 4 para-cancerous controls (Fig. 1h). By employing Flex scRNA-seq (10x Genomics) on these specimens, we expanded our investigation to confirm the intra-patient ITH (Fig. 1i). While SCLC-A and SCLC-N exhibited close relation, the NE-low subtype (SCLC-P) appeared more distinct (Fig. 1i).

**Fig. 2:**
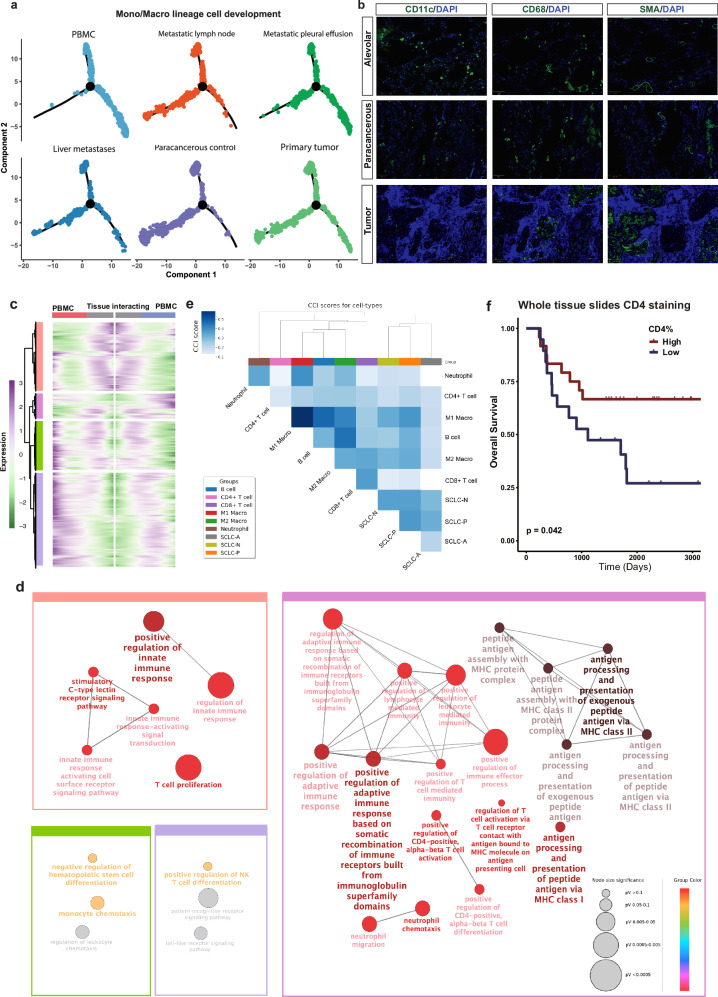
Myeloid lineage differentiation and TIME spatial location in SCLC. **a** DDRtree clustering of the Mono/Macro cells with DEGs selected using unsupervised methods implemented in monocle R package. **b** Representative immunofluorescence images showing the CD11c^+^ monocytes and CD68^+^ macrophages in the tumor vs stroma ROIs (based on SMA signal). **c** Heatmap visualizing genes that are differentially expressed in Mono/Macro cells from PBMC-enriched trajectories vs tissue-interacting trajectory. **d** Enriched gene ontologies of the DEGs in **c**. **e** CCI scores of each cell type pair based on the curated list of ligand–receptor (LR) pairs. Agglomerative hierarchical clustering was performed on a dissimilarity-like metric by taking the complement (1-score) of CCI scores, disregarding autocrine interactions. Cell types are colored by their lineages, as indicated in the legend. **f** Kaplan–Meier survival curves of 44 LS-SCLC patients based on the CD4 expression (stratified by median) assessed by IHC on whole tissue slides.

Given the significant presence and dynamic cancer–immune crosstalk observed, particularly involving Mono/Macro populations, we sought to trace the origin of specific Mono/Macro populations interacting with local cancer cells. As monocytes from PBMCs can differentiate into organ-specific lineages, we utilized pseudo-time trajectory analysis to identify transitional potentials within these populations. Our analysis revealed three distinct Mono/Macro lineages branching into various states, with two primarily PBMC-specific and a third monocyte population transitioning into a residential epithelium-interacting subtype (Fig. 2a). Subsequently, we identified differentially expressed genes (DEGs) enriched in this tumor-interacting subset. Compared to PBMC-enriched subsets, the TIME residential subset showed significant associations with T-cell activation, as well as concurrent MHC complex formation and peptide antigen presentation, suggesting a potential anti-tumor role co-regulated via the Mono/Macro axis in the SCLC TIME (Fig. 2c, d).

Based on the aforementioned conclusion, given that CD14^+^ myeloid cells can differentiate into Mono/Macro upon interaction with various cell types such as alveolar, cancer, and endothelial cells, and considering that macrophages are among the most abundant immune subsets capable of presenting MHC and activating T cells upon stimulation^24^, we proceeded to evaluate the distribution patterns of CD11c (monocyte marker) and CD68 (macrophage marker) using multiplex immunofluorescence. As anticipated, we observed a significantly higher infiltration of macrophages compared to monocytes within the SCLC microenvironment, indicating a locally adapted differentiation of monocytes into macrophages (Fig. 2d). These findings suggest myeloid adaptation and transformation into macrophages within the context of the SCLC microenvironment. Furthermore, we examined single-cell-level cell–cell communication by analyzing the crosstalk intensity between different cell types. Distance-based clustering revealed that aside from major immune cell interactions and the general tendency of an under-inflamed TIME in SCLC-A and SCLC-N subtypes, macrophages (tumor-associated and M2-like) were predominantly associated with the SCLC-P subset, indicating a yet-to-be-uncovered mechanism (Fig. 2e).

### DSP identifies cell composition and spatial gene regulatory networks associated with heterogeneity of NE features

Thus far, our scRNA-seq profiling has revealed the cellular heterogeneity between NE-high and NE-low cells in SCLC, along with the potential transformation of myeloid cells into macrophages within the local TIME. It is conceivable that the maturation of myeloid populations within the tissue milieu could lead to macrophage expansion and the recruitment of other immune cells, particularly after interacting with cancer cells. However, the intricate communication between tumor and stromal cellular components, as well as the spatial complexity of the TIME, cannot be fully elucidated using scRNA-seq alone. Therefore, we have employed DSP, an advanced ultra-high-plex transcriptomic and proteomic profiling technique, to dissect the complex spatial interaction within the SCLC TIME.

Since NE-high and NE-low SCLC are deemed to have distinct pathological features^25^, we initially assessed the histologic characteristics of 44 treatment-naive LS-SCLCs by quantifying T-cell infiltration levels, known to correlate with clinical benefit from ICI^26^. Through IHC, we evaluated the expression levels of CD3, CD4, CD8, as well as PD-L1 (both tumor proportion score (TPS) and combined positive score (CPS)). We found that higher CD4 level was associated with longer overall survival (OS) (Fig. 2f, *P* < 0.05), while CD8 and tumor-infiltrating lymphocyte (TIL) scores approached statistical significance (Supplementary Fig. S2, *P* = 0.051 and 0.083, respectively). Moreover, higher PD-L1 CPS scores conferred a greater survival benefit compared to PD-L1 TPS, underscoring the potential role of stromal-originated cellular components in determining clinical outcomes, as previously reported^27^.

**Fig. 3:**
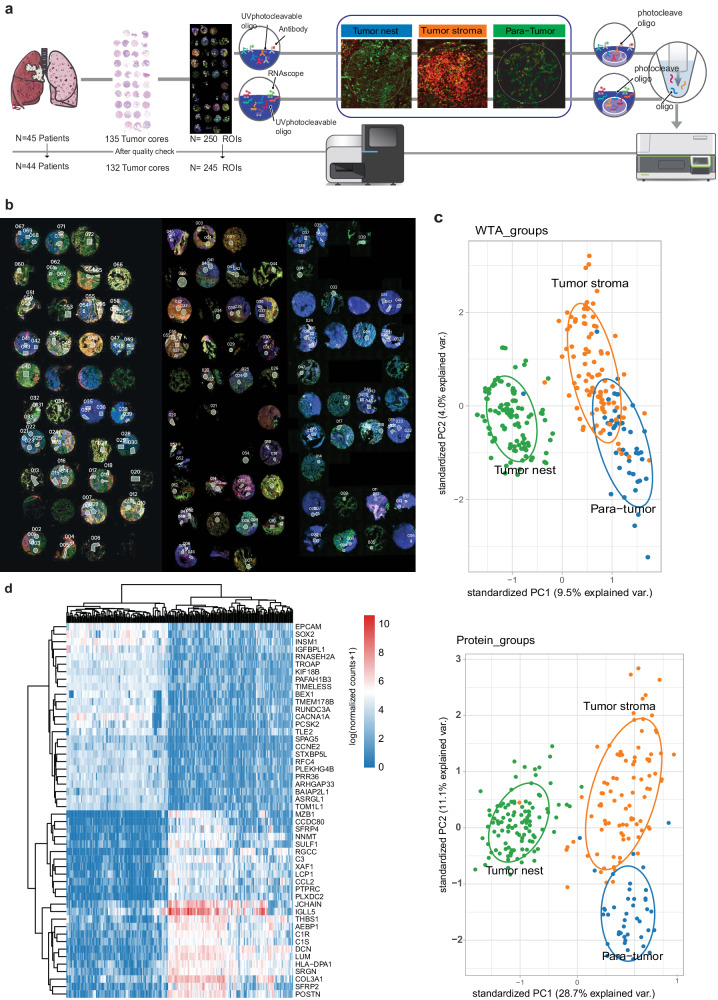
DSP profiles of immuno-oncological protein markers and whole transcriptomes from different pathological regions of SCLC. **a** Schematic of DSP profiling. Surgically resected tumors (*n* = 45) were subjected to DSP profiling. Three cores per tumor were selected by experienced lung cancer pathologists to build tissue microarray (TMA). One patient with final pathology showing large cell carcinoma was excluded. A total of 245 ROIs with different pathological characteristics were selected for DSP staining and subsequent analysis. Serial sections of the TMA were run through GeoMx RNA (top) or GeoMx protein (bottom) assays. Both assays were readout using next-generation sequencing. **b** Concomitant mIF images of TMA. Representative images for the tumor nest (inside tumor area and cytokeratin-positive), tumor stroma (inside tumor area and CD45^+^/CD3^+^ but cytokeratin^–^), and para-tumor region (tumor adjacent, morphologically normal lung) are shown. **c** PCA of WTA data (top) and protein data (bottom) from each ROI. Each dot indicates one ROI, and the *x* axis and *y* axis represent principal components 1 and 2, respectively. Green, orange, and blue colors represent tumor nest, tumor stroma, and para-tumor ROIs as defined above, respectively. **d** Differentially expressed immune-related genes annotated using GO_Immune dataset.

Upon validating our LS-SCLC cohort and recognizing the potential significance of T-cell levels and stromal cellular components in regulating the TIME, we proceeded to establish a comprehensive region-of-interest (ROI)-directed SPT landscape to uncover regulatory mechanisms within the multicellular context of SCLC (Fig. 3a, b). This analysis involved profiling 18,000 transcripts (whole transcriptome atlas (WTA)) and 60 key tumor-immune proteins across 245 ROIs, which included tumor-adjacent normal lung epithelium (hereafter termed para-tumor ROI), tumor cell-enriched areas (inside the tumor area and cytokeratin-positive; hereafter termed tumor nest ROI), and immune/T-cell-enriched areas (inside the tumor area and CD45^+^/CD3^+^; hereafter termed tumor stroma ROI), from the aforementioned 44 LS-SCLC tumors (Fig. 3b). Technically, following merging and normalization, both spatial whole transcriptome and proteome data exhibited clear separation of the three pre-defined spatial regions, thereby reinforcing the rigor of our results (Fig. 3c). While the tumor nest showed the lowest inter-ROI variance (WTA, xvar = 0.1122781, yvar = 0.3680549), protein expression spanned dynamically, exhibiting the highest inter-ROI heterogeneity (WTA, xvar = 0.1274768, Welch *t* test *P* value < 2.2e–16, yvar = 0.9755725, Welch *t* test *P* value = 7.747e–14; Protein, xvar = 0.1949548, Welch *t* test *P* value < 2.2e–16, yvar = 0.7971868, Welch *t*-test *P* value = 2.417e–06) (Fig. 3c).

**Fig. 4:**
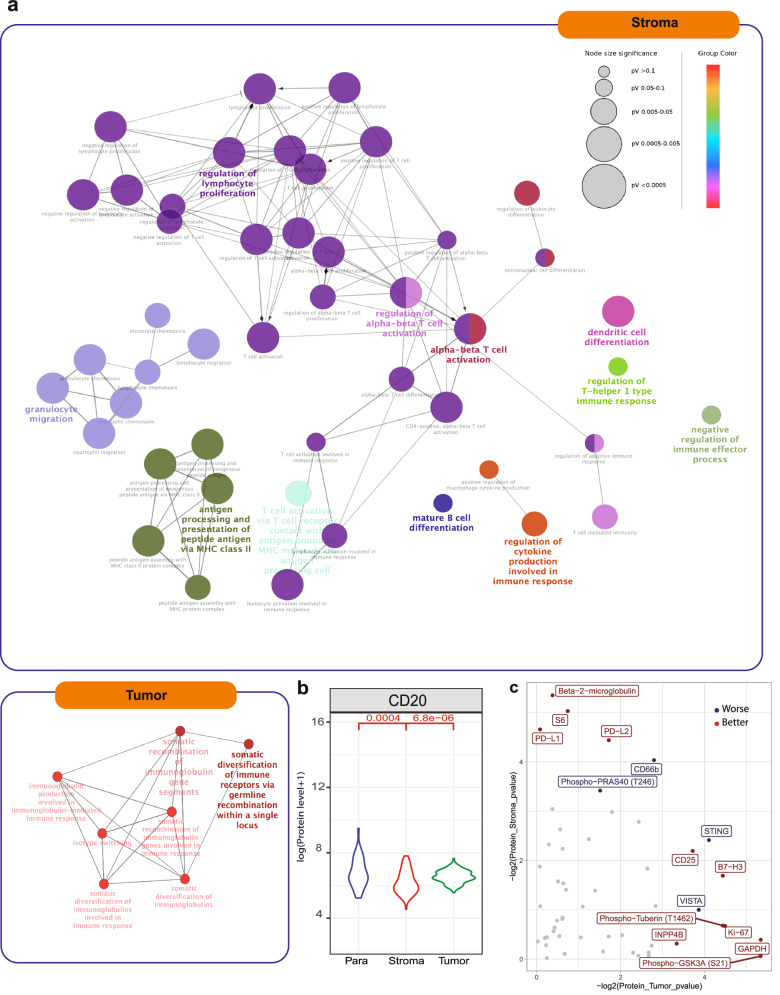
Enriched biological processes based on DSP profiles in different pathological regions of SCLC. **a** Enriched biological processes of tumor nest and tumor stroma ROIs. **b** The expression of CD20 protein in tumor nest (green), tumor stroma (orange), and para-tumor (blue) ROIs. **c** The protein levels in tumor stroma (*y* axis) and tumor nest (*x* axis) respectively associated with OS of 44 LS-SCLC patients. Red dots: proteins significantly (*P* < 0.05) associated with longer survival. Blue dots: proteins significantly (*P* < 0.05) associated with longer survival. The log-rank test was used for the survival analysis, and patients were stratified by the median level of each protein.

To gain deeper insights into intra-tumoral heterogeneity, we conducted a DEG analysis between the tumor nest and tumor stroma ROIs and elucidated their functional roles using Gene Ontology (GO) pathways (Figs. 3d, 4a). A subset of genes upregulated in the tumor nest were linked to immunoglobulin production and function (Figs. 3d, 4a). Conversely, the transcriptomic profiles specific to the tumor stroma were associated with diverse immune regulation and reprogramming processes, including T-cell expansion, active antigen processing and presentation, DC and B cell differentiation, macrophage-mediated cytokine production, and granulocyte translocation (Fig. 4a). Furthermore, our spatial proteomic data also indicated significantly higher B cell activity (marked by CD20^+^) in the tumor nest compared to the tumor stroma, underscoring the active antibody production originating from the tumor-enriched region (Fig. 4b). Notably, residential macrophages, as identified through our single-cell analysis, were found to be upregulated in SCLC but were predominantly associated with the tumor-surrounding stroma, as evidenced by higher levels of CD163 and CD80 present in the immune stroma (Supplementary Fig. S3). From a biomarker translation perspective, we systematically profiled differentially expressed proteins associated with OS. In immune stroma regions, higher levels of the total immune cell marker CD45, memory T-cell marker CD45RO, and cytotoxic immune marker GZMB were associated with longer OS, while higher expressions of neutrophil maker CD66b and hematopoietic stem cell marker CD34 were associated with shorter OS (Fig. 4c).

### Conventional NE markers and classifications show ITH of SCLC

**Fig. 5:**
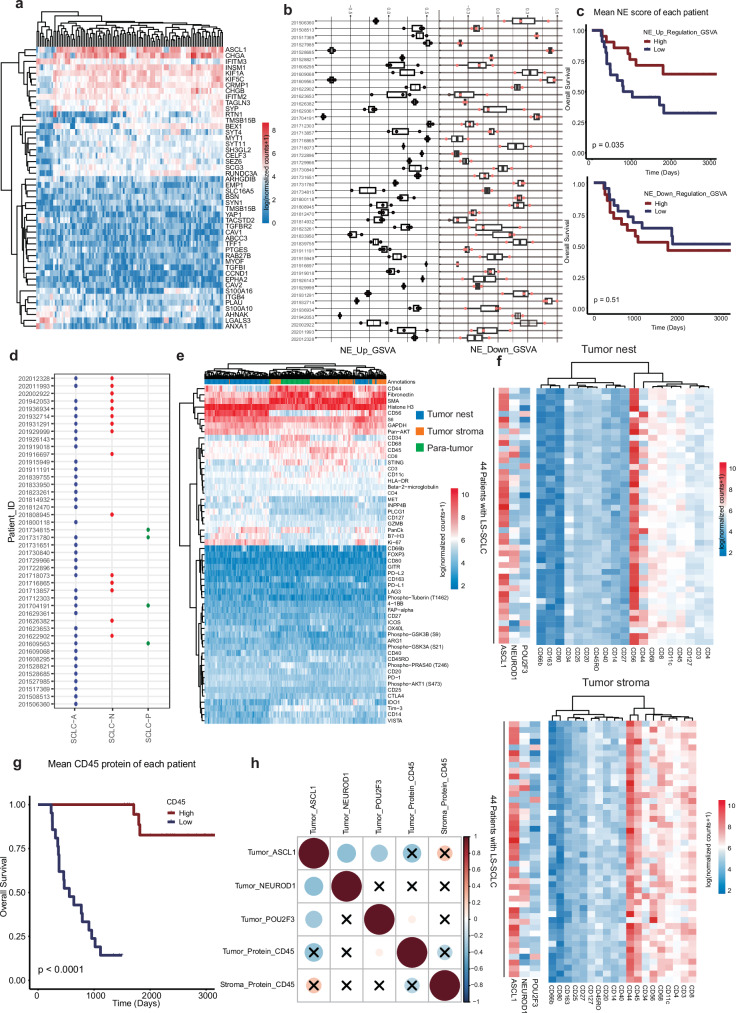
NE gene expression-based molecular subtyping and immune infiltration status. **a** The expression of 48 NE marker genes in all tumor nest ROIs. **b** The GSVA analysis of the NE marker gene expression of all tumor nest ROIs within each patient (NE scores up and down). **c** Kaplan–Meier survival curves of 44 LS-SCLC patients based on median cutoff point of NE scores. **d** Intra-tumor heterogeneity of previously defined SCLC molecular subtypes in different ROIs within the same LS-SCLC based on the expression of ASCL1 (SCLC-A), NEUROD1 (SCLC-N), and POU2F3 (SCLC-P). None of the tumors expressed YAP1 in this cohort. **e** Immuno-oncological and metabolism-related protein expression in tumor nest (green), tumor stroma (orange), and para-tumor (blue) ROIs. **f** The aggregated patient-level expression of molecular subtyping markers ASCL1, NEUROD1, POU2F3 (left) and the aggregated patient level of immuno-oncological protein markers in the tumor nest (top) and tumor stroma (bottom). **g** Kaplan–Meier survival curves of 44 LS-SCLC patients based on median cutoff point of CD45 protein levels. **h** Spearman correlation coefficients between the level of CD45 protein and expression of molecular subtyping markers.

NE features, as delineated by transcriptomic profiles from bulk sequencing, delineate the molecular subtypes of SCLC^18,28^. We next sought to define the molecular subtypes based on the WTA data collected from sub-histologically defined regions within these SCLC tumors. Initially, we extracted 48 key genes previously linked to SCLC NE differentiation^25^ and evaluated their expression across tumor nests using the WTA dataset. As anticipated, NE-high markers such as ASCL1, CHCA, KIF1A, RUNDC3A, BEX1, and TAGLN3 exhibited upregulation and co-clustering, while NE-low markers including YAP1, CAV1, ABCC3, MYOF, and RAB27B displayed general downregulation and were grouped together (Fig. 5a). Subsequently, we computed NE scores for each patient’s tumor at the ROI level, revealing significantly varied distributions of NE scores even among ROIs from the same tumors within both NE-high and NE-low groups, indicating ITH within individual tumors (Fig. 5b). Moreover, upon averaging the NE scores of ROIs within each patient, we observed that patients with lower NE scores were significantly associated with longer OS (Fig. 5c).

Recent studies have demonstrated that SCLCs of different molecular subtypes, as defined by expression of canonical markers (ASCL1, YAP1, NEUROD1, and POU2F3), exhibit distinct immune profiles and responses to immunotherapy^18,21^. We next classified the tumor nest ROIs based on the highest expression of these four classic SCLC subtyping markers. The ROIs were predominantly categorized into the SCLC-A subtype, with SCLC-Y not being identified in this cohort (Fig. 5d). Intriguingly, in 13 out of 43 cases, ROIs within the same tumors were designated as different molecular subtypes (patient 201916697 had only one tumor nest ROI, and hence was not included in this analysis), underscoring the significant ITH of NE features in SCLC and the limitations of SCLC subtyping from single biopsies.

Next, we explored the potential relationship between major immune cell markers derived from spatial proteomic profiles and SCLC molecular subtypes. Globally, tumor nest regions exhibited generally low immune expression, indicative of a cold tumor microenvironment (Fig. 5e). No significant association was observed between immune cell markers and SCLC subtypes (Fig. 5f), potentially due to the relatively small sample size, particularly in the immune-hot SCLC-P group. Although high expression of general immune cell marker CD45 expression was associated with improved OS (Fig. 5g), which is consistent with the results from CD45 protein DPS data, CD45 did not show significant association with any of these molecular subtype marker genes ASCL1, POU2F3, or NEUROD1 (Fig. 5h).

### Spatial tumor–immune proteo-transcriptomics pattern stratifies patients associated with different immune infiltration and NE features

**Fig. 6:**
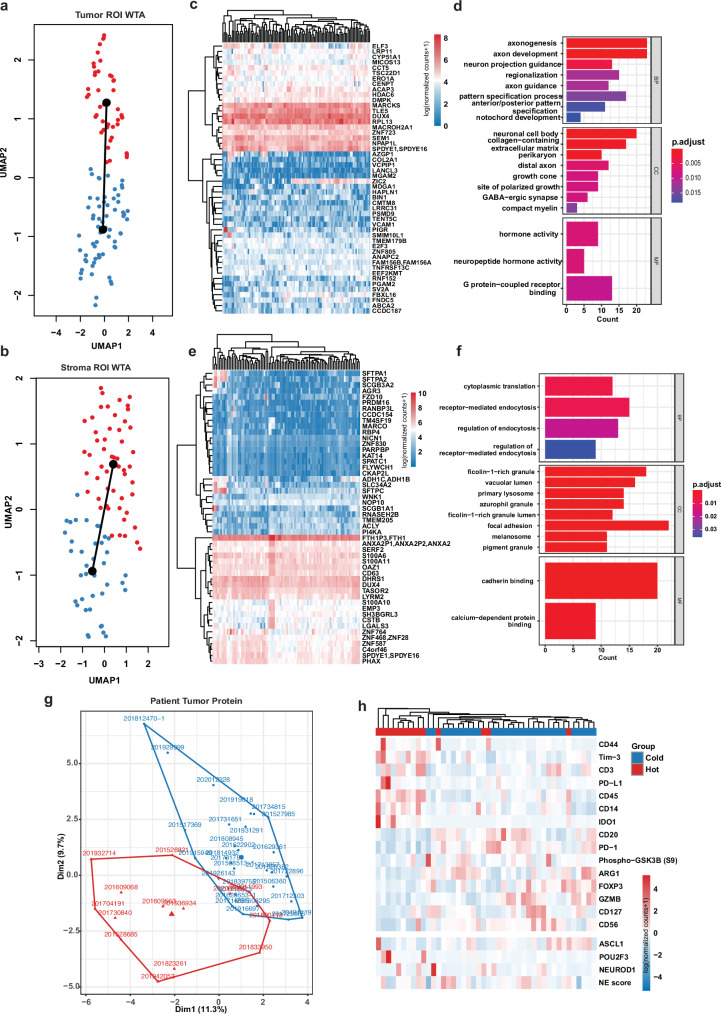
Heterogeneity of immune infiltration in tumor nest vs tumor stroma of SCLC. **a**, **b** UMAP dimensionality reduction plot visualizing WTA data from each tumor nest ROI (**a**) and each tumor stroma ROI (**b**). The colors of the dots represent clusters classified by Gaussian mixture modeling. **c**, **e** Genes that change with pseudo-time were visualized using heatmaps of their corresponding expression levels in tumor nest ROIs (**c**) and tumor stroma ROIs (**e**). **d**, **f** Enriched pathways of DEGs identified in tumor ROIs (**c**) and tumor stroma ROIs (**f**). **g** *K*-means clustering of protein levels in tumor ROIs aggregated by patient IDs. **h** Heatmap of proteins that are differentially expressed in two groups, identified using *K*-means clustering.

We then capitalized on the extensive WTA data to construct trajectories aiming to infer dynamic lineage changes during the development of SCLC and to identify genes driving this evolutionary process. Utilizing the Bayesian Information Criterion, we delineated the minimum spanning tree based on the ROI clusters to elucidate the global lineage architecture. Subsequently, we fitted the main curve to define each lineage, facilitating the identification of key genes associated with pseudo-time orders. This approach enabled the discovery of lineage-associated clusters in both tumor and stroma regions (Fig. 6a, b) and facilitated the identification of genes driving tumor development. Within tumor nests, these genes were primarily involved in extracellular matrix organization and cell projection assembly processes (Fig. 6c, d), while in the stroma, genes primarily involved in the regulation of endocytosis and focal adhesion were identified (Fig. 6e, f).

We next constructed a cross-region map for tumor nest, tumor stroma and para-tumor ROIs based on proteomics data using hierarchical clustering. Since we only have 60 protein targets from the DSP panel, we simply constructed a region-specific map based on these 60 proteins. By calculating the mean expression levels of individual proteins at each patient level, patients were overall grouped into two clusters defined roughly by their corresponding immune status (Supplementary Fig. S4a, b).

Furthermore, we applied *K*-means clustering to tumor regions across patients and also observed two main groups (Fig. 6g, h). One group exhibited relatively higher expression of immune checkpoints, including PD-L1, TIM-3, and IDO1, along with infiltrated CD3^+^ T cells and the monocytic marker CD14, defining it as an immune-hot. In contrast, the other group (immune-cold) showed elevated levels of the NE signal CD56, as well as increased expression of the Treg markers FOXP3 and CD127, highlighting a resistant or exhausted tumor microenvironment (Fig. 6h). Importantly, these findings aligned with the spatially defined molecular subtypes, particularly SCLC-A, which strongly correlated with an immune-deprived TIME in SCLC, as evidenced by the aforementioned transcriptomic profiles.

### Immune cell communication identifies the potential regulatory roles of myeloid cells in TIME of SCLC

**Fig. 7:**
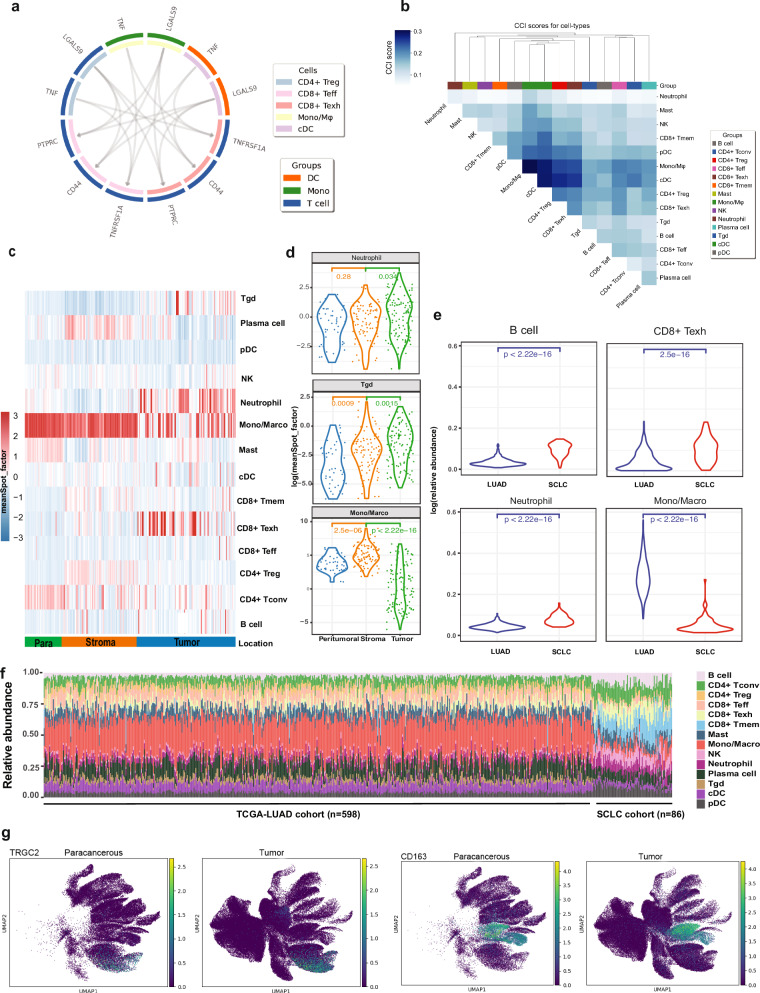
The innate immune response in TIME of SCLC. **a** The communication scores for each LR and cell pair. The lighter red color patches represent stronger interactions between sender and receiver cells. **b** CCI scores of each cell type pair based on the curated list of LR pairs. Agglomerative hierarchical clustering was performed on a dissimilarity-like metric by taking the complement (1-score) of CCI scores, disregarding autocrine interactions. Cell types are colored by their lineages, as indicated in the legend. **c** Heatmap showing the mean spot factor of the immune cell portion identified by the cell2location algorithm. **d** The abundance of major immune subsets in different spatial locations. Significance was assessed using the rank-sum test. **e** The abundance of major immune subsets in LUAD and SCLC. **f** Histogram showing the immune cell portion of SCLC and LUAD bulk RNA-seq identfied by cell-type deconvolution. The proportion of CD8^+^ Texh, Mono/Marco, B cells, and neutrophils in LUAD (left) vs SCLC (right). **g** UMAP plots showing expression of *TRGC2* and *CD163* in T cell subsets and macrophages, respectively. Plots represent para-tumor normal samples vs tumors and expression levels are shown in scaled on the right.

To further explore the immune regulatory mechanisms, we retrieved SCLC scRNA-seq data to examine intercellular crosstalk via cell–cell communication^23^. We calculated the nondirectional Bray-Curtis-like CCI scores for each immune cell pair. We observed communal ligand–receptor pairs present across multiple immune cell interactions (e.g., *MIF*/*CD74* and *CXCR4*), while some interaction pairs were present in specific cells (e.g., *HLA-DMA*/*CD4*) (Fig. 7a). Among the significant interactions, the *LGALS9*–*PTPRC*, *MIF*–*CD74*/*CD44*, and *TNF*–*TNFRSF1A* pairs were the most abundant interactions in the SCLC TIME (Fig. 7a; Supplementary Fig. S5). Importantly, mutual signals between Macro/Mono and *CD4*^*+*^ Tregs were observed, in line with potential functions of monocytes in driving macrophage transformation in the local TIME described above. These communications were likely mediated via the *CD86*–*CTLA4* and *CD99*–*PILRA* feedback loop (Fig. 7a; Supplementary Fig. S5). A reciprocal interaction was also noted between *CD8*^*+*^ T cells and plasma cells, which interacted via signaling through the *CD70*–*CD27* ligand–receptor pair (Fig. 7a; Supplementary Fig. S5).

We then proceeded to compile CCI scores and conducted distance-based clustering based on the interaction strength between the identified immune subsets (Fig. 7b). Consistent with our previous observations, antigen presentation via MHC class molecules mediated through the Macro/Mono/cDC axis exhibited the tightest interaction with *CD4*^*+*^ Treg and exhausted T cells, as indicated by closely clustered CCI scores within the TIME, highlighting their central regulatory roles in the SCLC TIME (Fig. 7b). Furthermore, we observed that *CD8*^*+*^ Texh, *CD4*^*+*^ Treg, monocytes, and plasma cells formed a co-regulatory cluster, suggesting potential interactions among these cell types (Fig. 7b).

We then validated these findings in the spatial context using cell2location-WTA to deconvolute cell subtypes in the TIME within each ROI. Interestingly, while the tumor stroma ROIs showed a higher overall degree of immune infiltration, *CD8*^*+*^ Texh, gamma delta T cells (Tgd), and neutrophils exhibited greater infiltration in tumor nest regions compared to tumor stroma or para-tumor ROIs (*P* < 0.05, Fig. 7d, e). Additionally, B cells were generally upregulated across tumor nest ROIs and tumor stroma regions compared to para-tumor ROIs (Fig. 7d).

We next sought to confirm whether the low Mono/Macro infiltration and high infiltration of neutrophils, B cells, and *CD8*^*+*^ Texh were unique to SCLC. Deconvolution of bulk RNA-seq data of TCGA lung adenocarcinoma (LUAD) vs a larger cohort of SCLC^21^ revealed significantly higher *CD8*^*+*^ Texh, B cells, and neutrophils while significantly lower Mono/Macro infiltration in SCLC than in LUAD (Fig. 7f, g, *P* < 0.05), consistent with our WTA results. Taken together, these results highlight the unique features of cold TIME in SCLC with overall low immune infiltration except *CD8+* Texh, B cells, and neutrophils, where the myeloid cells, including Mono/Macro, may play essential regulatory roles.

### REST is a novel marker in SCLC

**Fig. 8:**
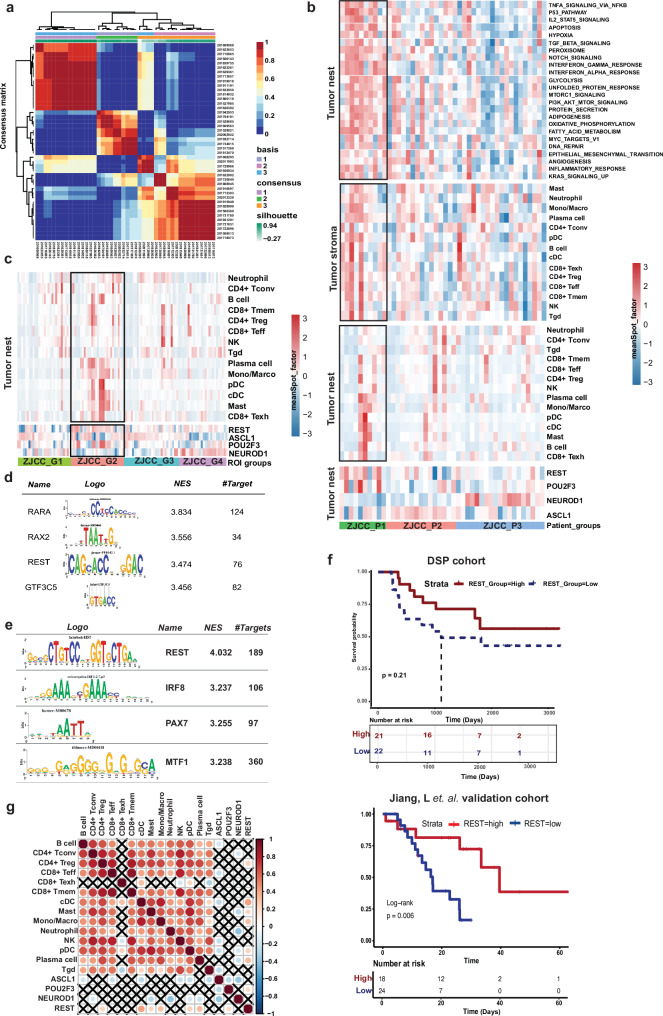
Identification of REST as a potential marker for NE-low SCLC associated with immune infiltration and patient survival. **a** The consensus map of the patients based on various statistical metrics decomposed by NMF. **b** GSVA enrichment fraction of the Cancer Hallmark processes in each patient, with 3 NMF-clustered groups represented (top). Relationship between the 3 NMF groups and immune cell infiltration in tumor stroma and tumor nests, respectively (middle two). Relationship between the 3 NMF groups and ASCL1, POU2F3, NEUROD1, and REST expression (Bottom). **c** Relationship between the four NMF subtypes and immune cell infiltration at ROI level. **d** Enrichment of transcription factors of potential regulatory genes that are differentially expressed, and clustered by patients. **e** Enrichment of transcription factors of potential regulatory genes that are differentially expressed, and clustered by ROIs. **f** Kaplan–Meier survival curves of LS-SCLC patients stratified by the REST expression level (at the optimal cutoff point) in the current DSP cohort and an external independent validation cohort of 42 patients. **g** Spearman correlation of the expression of previously defined molecular subtyping marker genes *ASCL1*, *POU2F3*, *NEUROD1,* and *REST* with infiltration of major immune subsets in all tumor nest ROIs.

To further delve into the molecular and cellular drivers associated with molecular classification in SCLC, we utilized non-negative matrix factorization (NMF) to extract key expression modules in tumor nest ROIs aggregated at the patient level. By using cophenetic correlation values to guide the selection of the optimal number of clusters, we identified clusters that aligned with the previously established molecular subtypes defined by ASCL1, NEUROD1, and POU2F3 (Fig. 8a). These clusters were then ordered, and major enriched pathways were extracted, along with tumor stroma ROI data aggregated at the patient level. Group 1 (ZJCC_P1) exhibited high POU2F3 expression (NE-low), while ZJCC_P2 and ZJCC_P3 showed higher ASCL1 and NEUROD1 expression (NE-high), respectively (Fig. 8b). Hallmark gene set enrichment via GSVA revealed that the ZJCC_P1 group was largely characterized by higher interferon responses and JAK-STAT signaling compared to the NE-high groups (ZJCC_P1 vs ZJCC_P2, *P* value = 0.07092; ZJCC_P1 vs ZJCC_P3 groups, *P* value = 0.00344). Additionally, compared to the NE-high groups, the NE-low ZJCC_P1 group also exhibited an active antigen-presentation characteristic, with numerically higher levels of monocytes and DCs, although this difference was not statistically significant, possibly due to the small sample size and high ITH within the TIME (Fig. 5d, Wilcoxon test, *P* value = 0.2858).

At the ROI level, tumor nest ROIs were clustered into 4 groups. ZJCC_G4 group had higher NEUROD1 expression (NE-high); ZJCC_G1 group and ZJCC_G3 group had higher ASCL1 expression (NE-high) and ROIs of ZJCC_G2 demonstrated high POU2F3 expression (NE-low) (Fig. 8c). Importantly, ROIs of NE-low ZJCC_G2 had significantly higher levels of monocytes (Wilcoxon test, *P* value = 8.852e–08), DCs (Wilcoxon test, pDC: *P* value = 0.001402; cDC: *P* value = 2.308e–06), and mast cells (Wilcoxon test, *P* value = 1.143e–05), suggesting that relatively immune-primed TIME in NE-low SCLC compared to NE-high SCLC (Fig. 8c).

Furthermore, we sought to identify transcription factors that may impact the differential expression of genes in POU2F3-high (NE-low) SCLC. Using motif analysis, we identified several regulatory candidates (Fig. 8d, e). Among these genes, REST was most consistent with the POU2F3-positive SCLC presentation in ZGCC_P1 (Fig. 8b, Wilcoxon test, *P* value = 0.008033). This was also proven by its expression across ROI classification and its high expression in ZJCC_G2 group at ROI level (Fig. 8c, Wilcoxon test, *P* value = 0.0001628). We cross-compared REST along with other canonical classification markers in the spatial expression data, and surprisingly, none of established SCLC subtyping markers ASCL1 (Wilcoxon test, ZJCC_P1 ~ ZJCC_P2, *P* value = 0.1151), NEUROD1 (Wilcoxon test, ZJCC_P1 ~ ZJCC_P2, *P* value = 0.3969) or POU2F3 (Wilcoxon test, ZJCC_P1 ~ ZJCC_P2, *P* value = 0.1438) was able to successfully discern NE-low patients (ZJCC_P1) from other two NE-high groups at the patient level, while REST clearly distinguished NE-low patients (ZJCC_P1) from NE-high patients (ZJCC_P2 and ZJCC_P3) (Wilcoxon test, *P* < 0.05) (Supplementary Fig. S6a). We further evaluated the correlation between the spatial expression of those subtyping markers and immune subsets based on the deconvolution of spatial transcriptomic data. None of the canonical molecular subtyping genes *ASLC1*, *NEUROD1,* or *POU2F3* was correlated with most of the immune cell infiltration, while expression of *REST* was positively correlated with multiple immune cell subtypes with the highest correlation with Mono/Macro and cDC (Fig. 8g). Importantly, the expression of REST was correlated with OS in both our DSP dataset (Fig. 8f, top) and an external validation set by Jiang, L. et al. (Fig. 8f, bottom)^29^, while none of ASCL1, NEUROD1 or POU2F3 was associated with survival in either our own DSP cohort (Supplementary Fig. S6b) or the external cohort^18^. Taken together, these results suggest that REST may be a superior marker for NE-low SCLC. Importantly, the tumor nest ROIs with high REST expression corresponded to tumor stroma ROIs with higher levels of T-cell infiltration and lower Mono/Marco infiltration (Supplementary Fig. S6c).

**Fig. 9:**
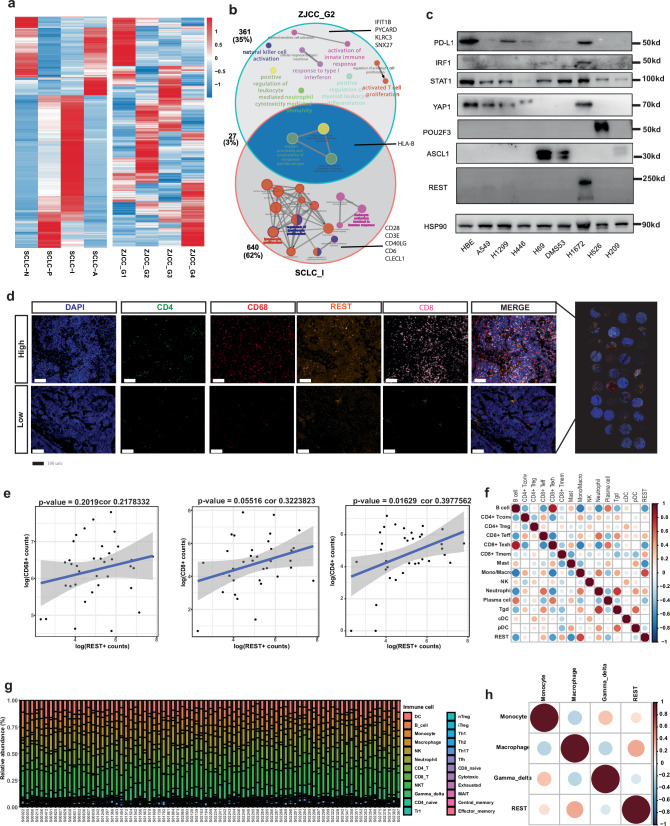
Upregulation of antigen presentation and alpha-beta T cells in regions with high REST expression. **a** The expression of the genes shared between SCLC-A/-I/-N/-P molecular subtyping panel and the 4 molecular subtyping panel established in the current study. **b** The shared and unique marker genes (top) and enriched pathways (bottom) in SCLC-I group and SCLC-REST group. **c** The western blotting quantification of PD-L1, IRF1, STAT1, YAP1, POU2F3, ASCL1, REST expression normalized to HSP90. **d** Representative mIF images of CD68, CD4, CD8, REST, and DAPI stainings. **e** Correlation between REST^+^ cancer cell density with CD4^+^, CD8^+^ and CD68^+^ immune cells identified by mIF. **f** The spearman correlation of *REST* expression and immune cell subsets derived by deconvolution of RNA-seq data from an external validation set. **g** Histogram showing the immune cell portion of SCLC and LUAD bulk RNA-seq identified by ImmuneCellAI. **h** The spearman correlation of *REST* expression and immune cell subsets derived by deconvolution of RNA-seq data from George et al. validation set.

Further, we investigated the SCLCs with high REST expression (SCLC-REST) in relation to a recently reported SCLC subtype with high immune infiltration termed SCLC-I^18^. The marker genes used to define SCLC-I or SCLC-REST were compared (Fig. 9a, b). Briefly, the genes with higher expression in SCLC-I than SCLC-A, -N or -P were defined as the markers for SCLC-I. A total of 27 genes were shared between SCLC-I and SCLC-REST subtyping panels, while 361 genes were unique in SCLC-REST panel and 640 were unique to SCLC-I subtyping panel (Fig. 9b). The marker genes shared by SCLC-I and SCLC-REST panels are involved in antigen processing and presentation highlighting the important roles of antigen processing and presentation on the TIME features of SCLC-I vs SCLC-REST subtypes. Genes unique to SCLC-I panel were enriched for lymphocyte activation and proliferation pathways, while the genes unique to SCLC-REST were related to the regulation of the innate immune activation (Fig. 9b). The heavier weight in innate immune activation and less lymphocyte activation/proliferation in SCLC-REST subtyping panel may reflect that SCLC-REST was based on tumor nest ROIs, while SCLC-I panel was based on bulk sequencing of SCLC tumors composed of both tumor nest and tumor stroma presumably. Finally, we validated REST as an SCLC immunological marker using an external SCLC scRNA sequencing dataset^30^. Within this dataset, an epithelial cluster characterized by higher *REST* expression was identified. Further analysis of cellular crosstalk revealed a tighter interaction between *REST*^*+*^ SCLC cells and immune cells compared to *ASCL1*^*+*^ or *NEUROD1*^*+*^ SCLC subtypes (Supplementary Fig. S7).

### REST-high SCLC has a more active immune response

Next, we aimed to investigate the potential role of REST in SCLC. We began by examining the expression of REST in established SCLC cell lines and its correlation with other immunogenic markers (Fig. 9c). Interestingly, while the REST^+^ cell line H1672 was distinct from ASCL1 and POU2F3 cells, it showed co-expression with YAP1 as well as PD-L1 and IRF1 (Fig. 9c). To further explore its function, we ectopically expressed REST in an ASCL1^+^ NE-high SCLC cell line H69 and observed downregulation of ASCL1 and HES1, validating its role as a suppressor of neural differentiation in SCLC (Supplementary Fig. S8). Subsequently, we used multiplex IHC to orthogonally validate our findings. Leveraging the same TMAs from the DSP experiment, we evaluated the expression of CD4, CD68, CD8, and REST, revealing a positive correlation between CD4^+^ T cells and REST^+^ cells (*R*^2^ = 0.39, *P* = 0.016) (Fig. 9d, e). Similarly, trends of positive correlation were observed between REST^+^ cells CD8^+^ cells, and CD68^+^ cells, supporting an overall pro-inflammatory function of REST in SCLC (Fig. 9e). Furthermore, we deconvoluted bulk RNA-seq data from previous studies^31^ to consolidate these findings. As anticipated, *REST* expression was positively correlated with *CD4*^*+*^ T conventional cells but not CD4^+^ Tregs. Additionally, *REST* expression was positively associated with Mono/Marco, *CD8*^*+*^ Teff, *CD8*^*+*^ Tmem, and negatively associated with B cells and *CD8*^*+*^ Texh, consistent with our WTA data, further supporting REST-high tumors as a pro-inflammatory subtype of SCLC (Fig. 9f). Further validation using ImmuneCellAI-based deconvolution on the dataset by George et al. revealed a positive correlation of monocytes or macrophages with REST, reinforcing its immune regulatory function within the SCLC TIME (Fig. 9g, h).

## Discussion

The aggressive nature of SCLC, as well as its diagnosis often occurring at the metastatic stage, results in a paucity of primary tumors for deep analysis, impeding our understanding of the disease. Previous studies focusing on SCLC tumor subtyping based on their NE features at either bulk or single-cell level have already shown promising potential in directing treatment stratification^18,25^ and many keynote studies are underway. On the other hand, gene expression-based classification is limited by the lack of spatial information that is key to underpin the bona-fide CCI and networking within the tissue microenvironment^23^. By incorporating both fresh samples and FFPE-archived tissues, our scRNA-seq profiling recaptured the previous NE-based molecular subtypes defined by ASCL1, NEUROD1, and POU2F3 but, more importantly, added a cellular transformation trajectory from peripheral blood immunity to local immunity highlighting potential roles of Mono/myeloid cell populations in regulating the TIME dynamics of SCLC, a finding also supported at protein level by our fluorescence in situ profiling. The relationship of immune–epithelial interplay differs across NE subtypes as revealed via our single-cell profiling, validating previous findings wherein NE-low tumors have a relatively higher association with multiple immune clusters, including macrophages (both M1- and M2-like), B cells as well as CD4^+^/CD8^+^ T cells. To gain deeper insights into the SCLC TIME, we constructed an ROI-defined SPT profile, an approach that allows greater potential to uncover critical mechanisms in deciphering the complex TIME. Our spatial profiling conducted on 44 LC-SCLC patients delineates vital differences happening across tumor nests, immune stroma, and para-tumor regions. Our spatial full transcriptome mapping not only chartered the tumor infiltration status of multiple immune cells whereby previously identified B cells, T cells subsets (CD4^+^ Treg/Tconv and CD8^+^ Tmem), monocytes, and macrophages via scRNA-seq are predominantly trapped within the tumor-surrounding regions but lowly embedded into the tumor nests. These findings are partially supported by previous studies indicating a lower T-cell infiltration, but rather diversified tumor clonality^12^. In addition to our scRNA-seq analysis based on multiple sites of SCLC where NE feature-related CCIs were observed, our comprehensive spatial molecular profile obtained at whole transcriptomic and key proteomic scales ensures sub-histological level discovery that was unmet through previous findings. Using pan-CK, CD3, and CD45 as morphological guidance to decompose TIME, our spatial analysis also successfully captured the molecular characteristics of SCLC with clear separation of three spatial compartments (tumor nests, immune stroma, and tumor-adjacent morphologically normal lung). Our spatial transcriptomic data extracted from tumors are robust enough to predict patient outcomes wherein NE-high tumor status implies a worse prognosis, a finding proven by previous studies. However, given the distinct molecular and immune features in compartmentalized TIME, NE-driven molecular subtyping defined from the homogenized tissues is inevitably insufficient and may somehow skew the biological understanding of SCLC. To prove this, by focusing on the tumor nest ROIs across patients, more than 30% of patients (13/43) had varying molecular subtypes as defined via conventional subtyping markers ASCL1, NEUROD1, and POU2F3 at ROI level even within the same tumor cores, highlighting the existence of ITH of SCLC. These ROI-derived data were also proven to correlate with distinct immune status as stratified by ASCL1, NEUROD1, and POU2F3. In particular, spatially derived protein expression profiles such as CD45 showed robust predictive capacity for patient stratification with survival benefit, again stressing the added value of spatial information for in situ biomarker discovery (*P* < 0.0001). Together, these data suggest the co-existence of multiple molecular subtypes within the same SCLC tumors that may significantly impact the predictive power of these molecular subtyping and certain spatially defined biomarkers may also warrant further studies at larger scales.

Of further importance, in addition to the above findings from spatial deconvolution analysis, our WTA data also indicate high CD8^+^ Texh, high Tgd cells, and high neutrophils enriched in SCLC tumor nests. CD8^+^ Texh cells are characterized by high PD-1, CD38, and TOX expression, and are mainly featured by loss of cytotoxic functions^32^. CD8^+^ Texh cells express factors that can actively recruit monocytes into local TIME and, in turn, polarize into residential macrophages, leading to exhaustion of CD8^+^ Teff during chronic antigen exposure^33^. Though macrophages are predominantly expressed in immune stroma regions, our WTA data did suggest a certain degree of macrophage infiltration within the tumor. This CD8^+^ Texh–macrophage interaction may explain the predisposed cytotoxic T lymphocyte exhaustion under a tumor-interacting context that is mediated via rising population of tumor-associated macrophages, a potential mechanism causing immunosuppression in SCLC.

As for Tgd cells upregulated in the tumor nests, since they are believed to impact the innate immune response rather than function via MHC-mediated antigen presentation^34^ their roles may involve other compensatory anti-tumor mechanisms that may be critical in the cold immune context of SCLC^35^. However, an alternative explanation for the high Tgd infiltration in SCLC is its failure to make the transition from innate to adaptive immune response, leading to impaired anti-tumor immunity. To address these, longitudinal studies may be needed to track the specific evolution path of SCLC.

Another interesting finding from WTA data is the elevated level of B cells within the tumor nest. This is further suggested by upregulation of immunoglobulin production pathways in tumor nests. These findings are in line with bulk RNA-seq data from published TCGA SCLC dataset wherein comparison between LUAD and SCLC via deconvolution methods implies higher ratio of B cells in SCLC^29^. In most cancer types, B cells involve in local tertiary lymphoid structure (TLS) formation, a prognostic indicator linking with better clinical outcomes^36^. However, in our data, TLS scores are relatively lower in tumor nests indicating tumor-enriched B cells may be dysfunctional to assist TLS formation. Furthermore, tumor nest expression of CD40 protein, another B cell marker, was shown to associate with worse OS in our cohort supporting the likelihood of these tumor-infiltrating B cells as being adverse in raising anti-tumor immunity in SCLC. Previously, SCLC is known to produce autoimmune antibodies that are associated with paraneoplastic syndrome^37^. Therefore, reverting B cell functionality may be another avenue to explore.

Our spatial profiling identified REST, a key regulator of a transcription factor that controls neuronal differentiation^38^, as a potential biomarker to define NE-low SCLC. REST functions by endogenously activating the Notch pathway, resulting in an NE to non-NE switch of tumor cells in a murine SCLC model and in humans^39^. A recent study has also shown the regulatory role of REST for NE to non-NE phenotype transition of SCLC that is mediated via the YAP/Notch/REST network^40^. REST has also been reported to impact metabolism, neurotransmitters, cytokines, and recruitment of immune cells, forming an inflamed TIME^41^. These are in line with our findings suggesting REST as being associated with more active immune/inflammation TIME and longer survival in SCLC and this was further validated by an external cohort^29^. Mechanistically, our spatial transcriptional data indicate its association with multiple immunomodulation pathways such as interferon production, interleukin response, and other inflammatory responses. Further, positive correlations of REST expression with multiple immune cells were observed based on spatial transcriptomic data as well as external RNA-seq data^29,31^. Interestingly, these were not found with previously reported subtyping markers, including ASCL1, NEUROD1, and POU2F3, suggesting its role as a superior biomarker for NE-low SCLC patient stratification and warranting future validation with independent cohorts.

Lastly, our study has several limitations. First, although our sample size is relatively large to establish a comprehensive ROI-directed SPT profiling of SCLC, it still remains small to conclude on certain aspects with statistical significance. Future studies on larger cohorts will be required to validate some important findings. Second, despite being carefully selected, the TMAs only represent a small portion of each SCLC tumor, a drawback that may underrepresent or overrepresent some of our findings, although multiple external validations were in place. Lastly, the ROI-based spatial profiling was incapable of reaching a true spatial single-cell level, impeding us from finding a mapping of cell fractions and their related mechanisms. Single-cell spatial analysis is indeed required to cross-validate some such as biomarker-specific cell types in future studies. Nevertheless, our SPT profiling at a sub-histological scale certainly made the progression of SCLC by uncovering unique immune features mapped to specific regions of SCLC, leading to some novel clues to be followed in the future. To harness specific cell types within the TIME may potentiate novel therapeutic strategies to be implemented and those include reverting B cell functionality, leveraging on innate immune system to facilitate the transition from innate immune response to adaptive immune response to improve clinical efficacy. In addition, the discovery of REST as a novel and potential biomarker to define NE-low patients may lead to biomarker-stratified clinical regimes such as ICIs.

## Materials and methods

### Patient cohorts and specimens

This study was approved by the institutional review board of the Zhejiang Cancer Hospital. 19 fresh samples (primary tumors, metastases, and PBMCs) from three patients with SCLC and two patients with LCNEC were obtained for scRNA-seq using 10x Chrominium technology. Meanwhile, FFPE specimens of 16 SCLC tumors and 4 para-tumor lung tissues were collected for 10x Flex scRNA-seq. The DSP study cohort comprised 44 individuals with LS-SCLC who underwent surgical removal. Sample collection was approved by the institutional review board of the Zhejiang Cancer Hospital with written informed consent from all patients included in the study. Clinical data including age, gender, smoking history, tumor stage, treatment history, and information on samples of all patients were reported in Supplementary Table S1.

All tumor samples were pathologically evaluated by two independent expert pathologists who inspected the histomorphology based on hematoxylin and eosin (H&E) and IHC staining of TTF-1, Sy, CgA, and CD56. H&E and IHC images of 10 cases of SCLC and 2 cases of LCNEC were shown in Supplementary Fig. S9. The remaining FFPE tissues were used to generate TMAs at 5-μm thickness. TMAs used for downstream analysis were reviewed by a senior clinical pathologist using H&E staining to ensure that representatives of the tumor, stroma, and peri-tumor were retained for individual cores. For each patient, three independent cores representing the tumor, adjacent stroma, and distal peri-tumor (1.5 mm in diameter) were chosen, yielding a total of 132 cores for IHC and high-plex spatial profiling.

### Immune marker quantification in SCLC patients

Primary antibodies against human CD8 (Clone 4B11, Cat# PA0183), CD4 (Clone 4B12, Cat# PA0427), and CD3 (Clone LN10, Cat# PA0553) were purchased from Leica (Leica Biosystems, Newcastle, UK). Antibody staining was visualized using the recommended BondTM Polymer Refine Detection kit (Leica Biosystems). PD-L1 IHC staining was performed using a 22C3 pharmDx kit (Cat# SK006; Dako Inc.). All primary antibodies were prepared and used according to the manufacturers’ instructions. Positive PD-L1, CD3, CD4, and CD8 staining was defined as any distinct and complete linear cell membrane staining, with or without plasma staining. The median levels for CD3, CD4, and CD8 were set as the cutoff values in the subsequent survival analyses.

### Immunofluorescence staining and screening of tissue slides

Slides were loaded onto glass slide holders and dewaxed with gradient of xylene and ethanol for 10–20 s each. Slides were washed with running tap water and then transferred to pre-warmed water (94–96 °C) containing an antigen retrieval solution (EDTA, pH = 9.0). The slides were then washed again under running water. Target antibodies were added at appropriate dilutions and time (1:50, 1:100, 1:200, etc.) for immunostaining. After PBS wash, secondary antibodies (2 μg/mL each) were added to the sections. Control experiments were conducted to ensure there was no cross-reactivity between antibodies.

### SPT analysis using digital spatial profiler

In general, TMA slides were deparaffinized with xylene and rehydrated in gradient concentrations of ethanol. Antigens were heat-retrieved either by ethylene diamine tetra-acetic acid (Tris-EDTA, pH 9.0) for RNA profiling, or citrate buffer for protein profiling. For RNA profiling, tissues were pre-digested with protease K (Thermo Scientific) to allow efficient RNA exposure prior to probe hybridization of over 18,000 genes in the WTA panel (NanoString). For protein detection, tissues were immediately blocked and incubated with 60 panelized detection antibodies. Both workflows included a morphological staining procedure using a cocktail of fluorescently labeled antibodies targeting pan-CK for tumor cells, CD45 for immune cells, and CD3 for T cells. Slides were then scanned on the DSP system, generating tri-colored images to guide ROI selection within the cores. One or two ROIs containing more than 100 cells were selected for each core. The cellular contents of each ROI were assessed and annotated as tumor-enriched, immune stroma, or peritumoral region under pathological assistance. Following ROI selection, photocleavable oligonucleotides (barcodes) for individual targets were UV-cleaved, collected, and quantified using either next-generation sequencing (Illumina NovaSeq 6000) or a nCounter system (NanoString) for RNAs and proteins, respectively. The generated raw data were subsequently demultiplexed on the DSP system to generate a count matrix for downstream analysis.

### DSP-WTA data quality control and normalization

The limit of detection was set as the geometric mean of the negative probes for each ROI according to manufacturer’s recommendations. Genes with counts below the detection threshold in each ROI were omitted from further analysis. Quantile analysis (Q3 norm) was used to normalize the data, and the limma package was used to eliminate the batch effect.

### DSP protein quality control and normalization

The digital counts of antibodies were divided by the geometric mean of the three IgG negative control antibodies on a per-ROI basis to establish the signal-to-noise ratio (SNR) as described below.

$${SNR}={Input}/\root{3}\of{{}\left({MsIgG}1* {MsIgG}2a* {RbIgG}\right)}$$

Antibodies in all ROIs below an *SNR* of one were considered lower than normal condition. Six normalization approaches were performed and cross-compared. Protein signals between ROIs were normalized using the quantile. Sample phenotypic groups were visualized using classical (multi-dimensional scaling) plot. The homogeneity and heterogeneity of samples from the same patient were visualized using boxplots. Analyses were performed using R (version 4.2.0) and RStudio (version 1.3.1093).

### Differential gene expression and functional enrichment of DSP-WTA data

WTA expression profiles were analyzed using the limma package. Downstream enrichment analysis was performed using GO (a community-based bioinformatics resource) and Kyoto Encyclopedia of Genes and Genomes (KEGG, an integrated database resource for the biological interpretation of multi-omics data; ClusterProfiler (R package) and ClueGO (CytoSCAPE plug-in) were used to examine GO and KEGG pathway enrichment.

### Spatial deconvolution of DSP-WTA data

Lineage estimation and quantitation of immune cells in each ROI were performed using the default SpatialDecon and Cell2location-WTA package optimized for DSP data^42^. For SpaitlalDecon-based cell deconvolution, reference cell type signature matrix was generated using snRNA-seq from SCLC patients published by Chan JM et al. as ref. ^23^. Patients treated with chemotherapy and immunotherapy were purposely excluded from the study. Raw WTA count matrix with relevant cell counts (determined by nuclei) was used as input data.

### Inferring immune CCI using DSP-WTA data

The Cell2cell package was used to compute CCIs^43^. Human LR pairs and the SCLC immune cell scRNA-seq expression matrix were used as input data. The CCI score was defined as the percentage of ligands produced by one cell that interacts with cognate receptors in another cell.

### LR correlation across ROIs calculated using WTA data

Known LR pairs were obtained from CellPhoneDB v2.0, and correlations between potential LR pairs were quantified using Spearman rank correlation between paired segments within the same ROI type and across all ROIs with the stated pairs. Statistical significance was set at *P* < 0.05.

### Collection of NE lung cancer samples and preparation of single-cell suspensions

Primary tumors, metastatic lymph nodes, metastatic pleural effusions, PBMCs, liver metastatic tissue, and para-cancerous controls from three SCLC and two LCNEC patients were used. All samples were obtained during surgical biopsies, digested, and physically dissociated into single-cell suspensions with cell viability over 85% as a passing criterion for downstream scRNA-seq analysis. The samples were cryopreserved as single-cell suspensions in RPMI medium containing 20% fetal bovine serum and 10% dimethyl sulfoxide. The single-cell suspension used for scRNA-seq was washed twice with PBS containing 0.04% BSA, and the final cell concentration was adjusted to ~1000 cells/μL. PBMCs were then sorted using a flow cytometry sorter to separate immune (CD45^+^) and non-immune cells (CD45^–^).

### Single-cell RNA library construction and sequencing

Sorted cellular proportions (CD45^+^ and CD45^–^) were loaded to 10x Chrominium, respectively. A pool of ~750,000 barcodes is sampled separately to index each cell’s transcriptome. It is done by partitioning thousands of cells into nanoliter-scale Gel Beads-in-emulsion (GEMs), where all generated cDNAs share a common 10x barcode. Libraries were generated and sequenced and 10x barcodes were used to associate individual reads back to the individual partitions (Shanghai Genechem Co., Ltd).

### 10x Flex data preprocessing, batch effect removal, and cell type annotation

The Illumina Hiseq PE150 readout was processed by the cellranger (10x Genomics). The Scanpy was employed for the cell filtering, normalization, clustering, and subsequent analysis. Specifically, the mitochondria percentage was jammed at 20%, the gene number was selected between 500 to 7000, and the gene was recognized as highly confidential with the observation in at least 10 cells. In current analysis, from 16 patients with SCLC and 4 SCLC para-cancerous tissue samples, we obtained 189,717 cells, after filtering, 150,252 cells remained. The cells were normalized, and the highly variable genes were selected. For multiple samples, the batch effect was removed by the BBKNN algorithm. The UMAP was employed for visualization, and the Louvain/Leiden with optimal resolution was used for clustering. The cell type annotation was initially given by CellTypist with immune cells and human lung cell atlas as references. The CellTypist results were manually checked with known marker genes retrospectively.

### NMF-based cluster identification

NMF algorithm was used to analyze the genes selected with the following criteria: 1) gene expression bimodal index > 1.5, 2) mean expression value > 25th percentile, and 3) standard deviation of expression value > 50th percentile. The cophenetic correlation coefficient was used to select the optimal cluster number for subsequent analysis. For SCLC samples, molecular subtype assignment was defined by the highest expression of three known transcription factors (*ASCL1*, *NEUROD1,* and *POU2F3*).

### Cell type annotation and pseudo-time inferring using scRNA-seq data

Preprocessed data were subject to integration, normalization and clustering using the Seurat package (v4.4.0). The dimensional reduction was performed using unsupervised UMAP considering the top 10 computed principal components with a resolution set to 1.2. The number of principal components used in the UMAP cluster was determined by taking the standard deviations of the top 20 principal components and running a jackstraw analysis to quantify the *P* value distributions^44^. Cluster marker genes and DEGs were identified using Wilcoxon rank-sum test. The differential expression between clusters applied a threshold of 0.25 for log2-fold change and a filter of minimum percentage of cells in a cluster greater than 25% as standards. Differentially expressed marker genes between clusters were identified by comparing significantly upregulated genes, defined as those with adjusted *P* < 0.05, and unique, non-shared genes between clusters.

Annotated cell types were obtained from the Satija lab PBMC dataset^45^, which was taken as the anchor reference Anchors searched between the reference and our query sets. Cell-type labels were then transferred from the reference set to the query set. Cell trajectory and pseudo-time analyses were performed using the PAGA algorithm in the Monocle R package (v2.8.0) and the reverse-graph embedding machine learning algorithm^46^.

### Identification of transcription factor regulators

A PPI network of DEGs was established using the STRING database (http://string-db.org) and visualized using Cytoscape, an open-source bioinformatics and graphic interface. The Cytoscape plug-in iRegulon^47^ was used to analyze transcription factors that regulate the hub genes. Transcription factor information was obtained from databases such as Transfac, Jaspar, Encode, Swissregulon, and Homer, which use genome-wide ranking and recovery to detect enriched transcription factor motifs and optimal sets of their direct targets. The cutoff criteria were: enrichment score threshold = 3.0, ROC threshold for AUC calculation = 0.03, rank threshold = 5000, minimum identity between orthologous genes = 0, false discovery rate (FDR) = 0.001, and normalized enrichment score (NES) > 3.

### Public data acquisition

RNA-seq and clinical data from 99 SCLC patients^29^ and patients with LUAD (TCGA) cohort were used. In SCLC cohort, 86 RNA-seq samples were obtained. Among them, 79 samples were tumor (purity > 70%), and 7 samples were para-tumor samples. The fragments per kilobase of exon model per million mapped fragments data were normalized, and the batch effect was removed. SCLC cell line metabolomic and proteomic data were obtained from the depmap project (https://depmap.org/). The dataset used for Chen, Y. et al.’s study was also analyzed to study the gene expression correlation^21^.

### Bulk-seq data integration and immune cell deconvolution

Processed gene expression data were scaled and normalized using the limma package in R (version 3.5.2). To quantify the abundance of immune cell infiltration, we employed TAPE^48^, an accurate cell-type deconvolution and gene expression analysis tool. The single-cell references were used as above. The model was trained with a batch size of 128 for 128 epochs.

### 10x Flex scRNA-seq from the FFPE achieved SCLC patient samples

FFPE specimens of 16 SCLC tumors and 4 para-tumor lung tissues from 16 patients were subjected to 10x Flex scRNA-seq as previously described^49^. In brief, two 25-µm FFPE sections from each specimen were obtained with a manual rotary slicer (HistoCore BIOCUT, Leica). Paraffin was removed by xylene and gradient ethanol. The tissue was then dissociated with a dissociation enzyme and pestle (10x Genomics). The human probe set for the 10x Genomics single-cell gene expression flex fixed RNA profiling assay was used to probe the targeted protein-coding genes. The Chromium was used to construct GEMs for sequencing pools (experiments were conducted by Cosmos Wisdom Biotech, Hangzhou, China).

### Cell lines, REST-pCDH vector construction, overexpression, and western blotting assay

All cell lines were sourced from ATCC. The cell lines were cultured in RPMI1640 supplemented with 10% fetal bovine serum (Gibco) and 1% penicillin/streptomycin. The cultures were kept at 37 °C with 5% CO_2_. For transient overexpression, *REST* cDNA was inserted into pCDH vector, cloned and purified REST-pCDH was verified by sequencing.

SDS-PAGE was used to assess REST-related protein work. In brief, membranes were blocked in 5% milk or 3% BSA, and followed by primary antibody incubation overnight at 4 °C. Membranes were then incubated for 2 h at room temperature with the HRP-conjugated secondary antibody (Abcam) and developed with an HRP peroxide reagent (Abcam).

### Statistical analysis and reproducibility

No statistical method was used to determine the sample size for power analysis. Unless stated otherwise, *P* values of 0.05 were deemed as a statistical significance cutoff. FDR was used for multiple correction adjustment, and 0.05 was chosen as a cutoff. For comparison between unpaired data groups, Kruskal walls and Wilcoxon were used, treating data as with non-normality with unequal variance.

## Supplementary information

Supplementary Information

## Data Availability

The raw DSP data used in this paper have been uploaded to the Genome Sequence Archive of the National Genomics Data Center, China National Cancer for Bioinformation (CNCB), Chinese Academy of Science (CAS), and are accessible, with restriction, at https://ngdc.cncb.ac.cn/gsa under the accession number HRA004312. Personal patient information and pathological images can be obtained with reasonable scientific request and institutional approval from the corresponding author. The customized code used for the analysis is available from the corresponding author at reasonable scientific request.
