# Author Correction: Single-cell and spatial proteo-transcriptomic profiling reveals immune infiltration heterogeneity associated with neuroendocrine features in small cell lung cancer

**DOI:** 10.1038/s41421-024-00755-z

**Published:** 2024-12-19

**Authors:** Ying Jin, Yuefeng Wu, Alexandre Reuben, Liang Zhu, Carl M. Gay, Qingzhe Wu, Xintong Zhou, Haomin Mo, Qi Zheng, Junyu Ren, Zhaoyuan Fang, Teng Peng, Nan Wang, Liang Ma, Lungevity PANSHI Initiative Consortium, Qingzhe Wu, Nan Wang, Yuefeng Wu, Hai Song, Yun Fan, Hai Song, Jianjun Zhang, Ming Chen

**Affiliations:** 1 Zhejiang Cancer Hospital, Hangzhou Institute of Medicine (HIM), Chinese Academy of Sciences, Hangzhou, Zhejiang China; 2 Zhejiang Key Laboratory of Radiation Oncology, Hangzhou, Zhejiang China; 3 The MOE Key Laboratory of Biosystems Homeostasis and Protection, Zhejiang Provincial Key Laboratory for Cancer Molecular Cell Biology and Innovation Center for Cell Signaling Network, Life Sciences Institute, Zhejiang University, Hangzhou, Zhejiang China; 4 School of Life Sciences, Westlake University, Hangzhou, Zhejiang China; 5 Department of Cardiovascular Surgery, The First Affiliated Hospital, School of Medicine, Zhejiang University, Hangzhou, Zhejiang China; 6 Zhejiang University-University of Edinburgh Institute (ZJU-UoE), School of Medicine, Zhejiang University, Haining, Zhejiang China; 7 Department of Thoracic/Head and Neck Medical Oncology, Division of Cancer Medicine, The University of Texas MD Anderson Cancer Center, Houston, TX USA; 8 College of Information Science and Electronic Engineering, Zhejiang University, Hangzhou, Zhejiang China; 9 Cosmos Wisdom Biotech Co. Ltd., Hangzhou, Zhejiang China; 10 Center for Oncology Medicine, The Fourth Affiliated Hospital of School of Medicine, and International School of Medicine, International Institutes of Medicine, Zhejiang University, Yiwu, Zhejiang China; 11 Department of Genomic Medicine, Division of Cancer Medicine, The University of Texas MD Anderson Cancer Center, Houston, TX USA; 12 State Key Laboratory of Oncology in South China, Guangdong Key Laboratory of Nasopharyngeal Carcinoma Diagnosis and Therapy, Guangdong Provincial Clinical Research Center for Cancer, Sun Yat-sen University Cancer Center, Guangzhou, Guangdong China; 13 United Laboratory of Frontier Radiotherapy Technology of Sun Yat-sen University & Chinese Academy of Sciences Ion Medical Technology Co., Ltd, Guangzhou, Guangdong China; 14 Westlake Laboratory of Life Sciences and Biomedicine, School of Life Sciences, Westlake University, Hangzhou, Zhejiang China

**Keywords:** Cancer microenvironment, Small-cell lung cancer

Correction to: *Cell Discovery* 10.1038/s41421-024-00703-x, published online 04 September 2024

In the original publication of this paper^1^, the accession number for the raw sequencing data was incorrect. The raw DSP data generated in this study have been uploaded to the Genome Sequence Archive of the National Genomics Data Center, China National Cancer for Bioinformation (CNCB), Chinese Academy of Sciences (CAS), and are publicly accessible at https://bigd.big.ac.cn/gsa-human/browse/HRA003705 under the accession number HRA003705. This correction does not affect the description of the results or the conclusions of this work.
