# Supplementary material for: Single-cell and spatial proteo-transcriptomic profiling reveals immune infiltration heterogeneity associated with neuroendocrine features in small cell lung cancer
Source: Cell Discov. 2024 Sep 4;10:93. doi: 10.1038/s41421-024-00703-x (PMC11375181; doi:10.1038/s41421-024-00703-x)
Supplement: Supplementary file 1 — Supplementary Information [file 41421_2024_703_MOESM1_ESM.pdf]

Supplementary Fig. S1

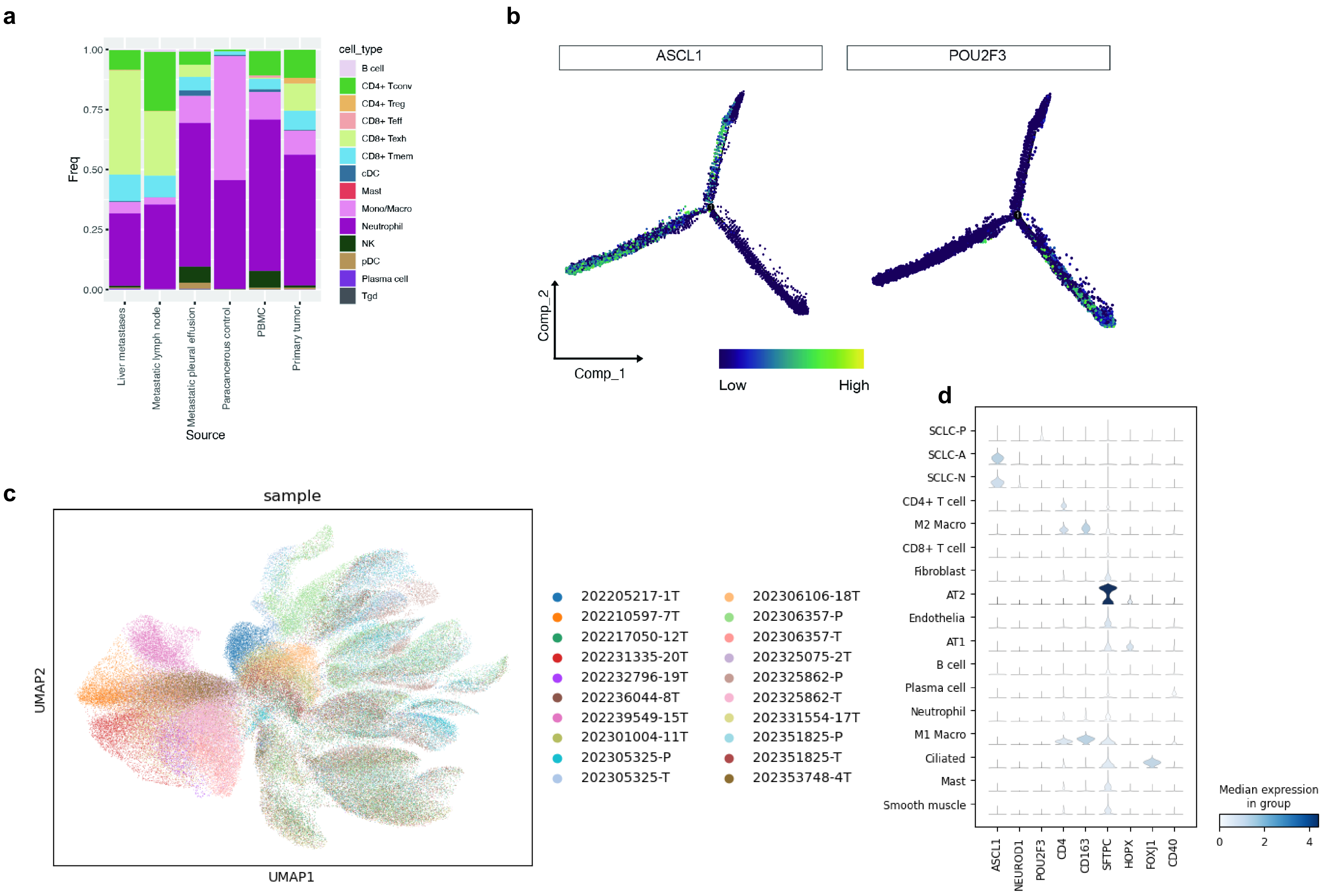

Supplementary Fig. S2

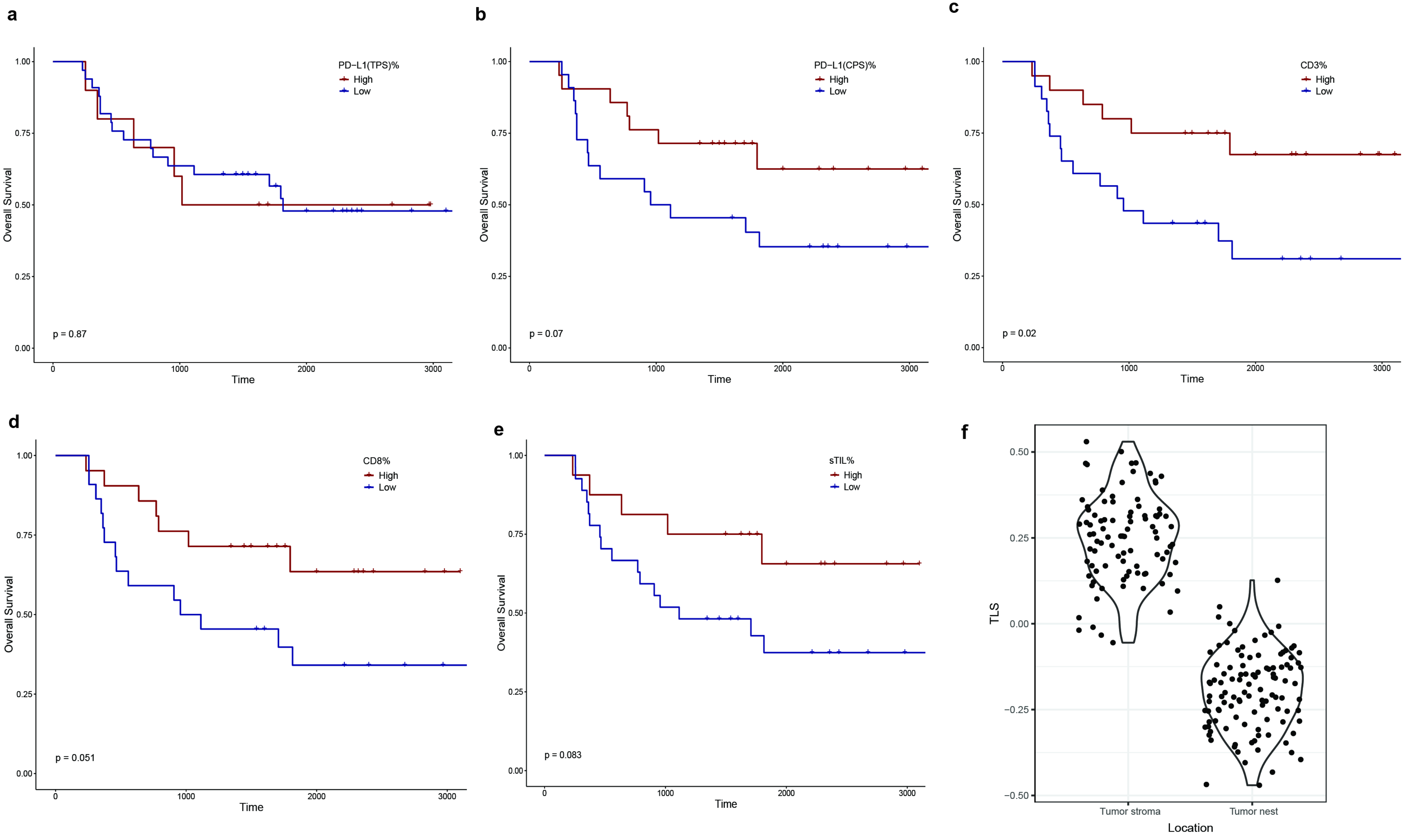

Supplementary Fig. S3

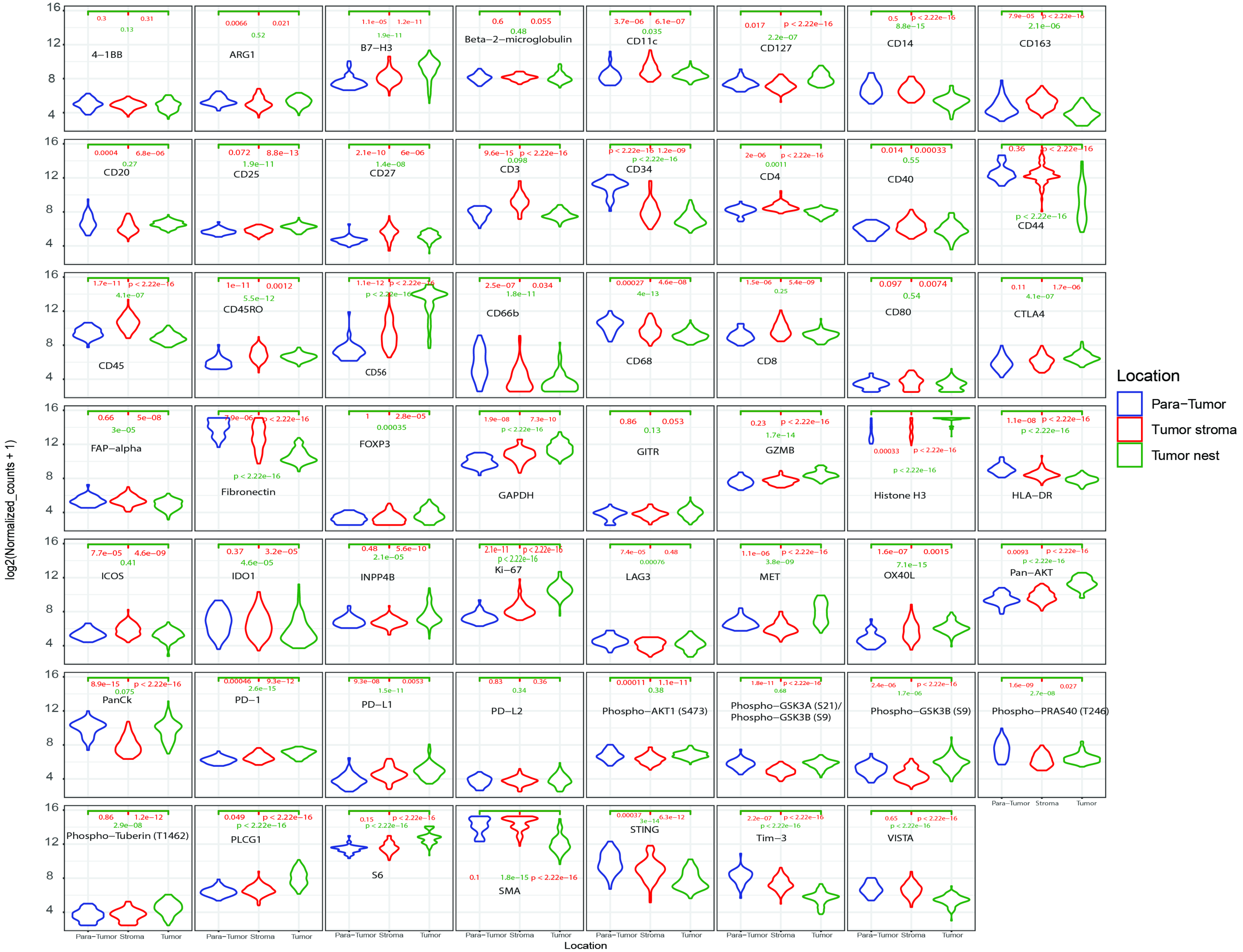

Supplementary Fig. S4

a

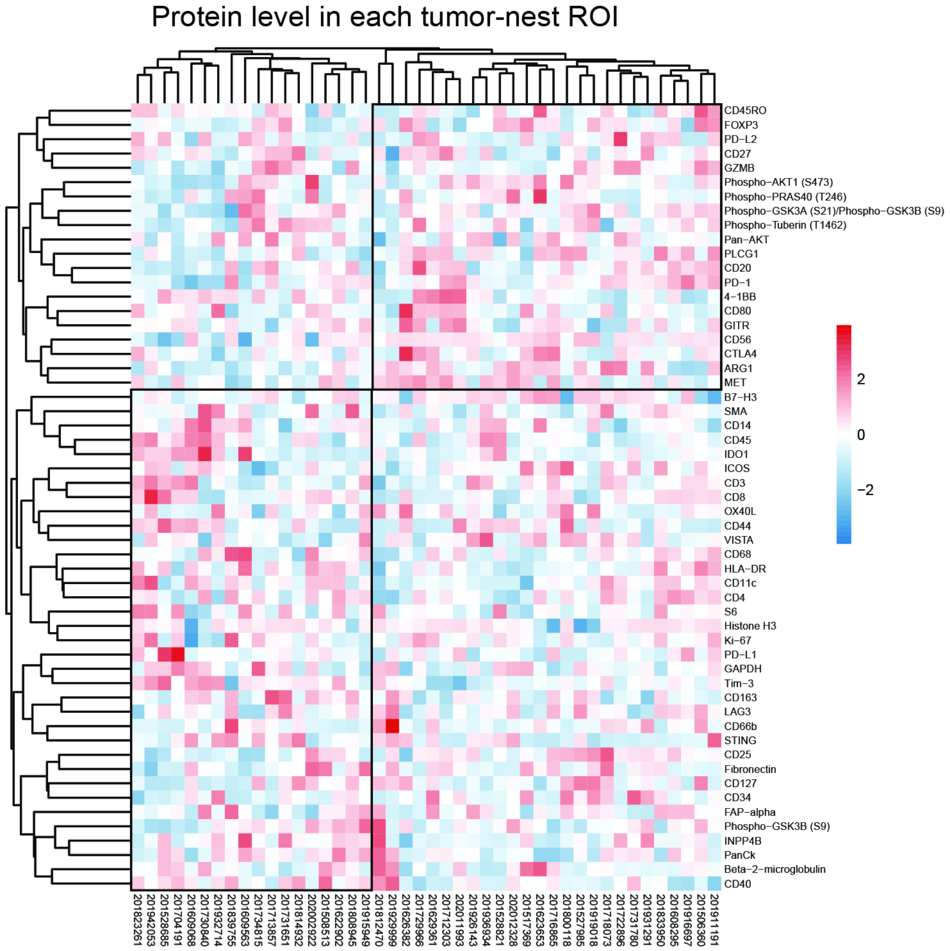

b

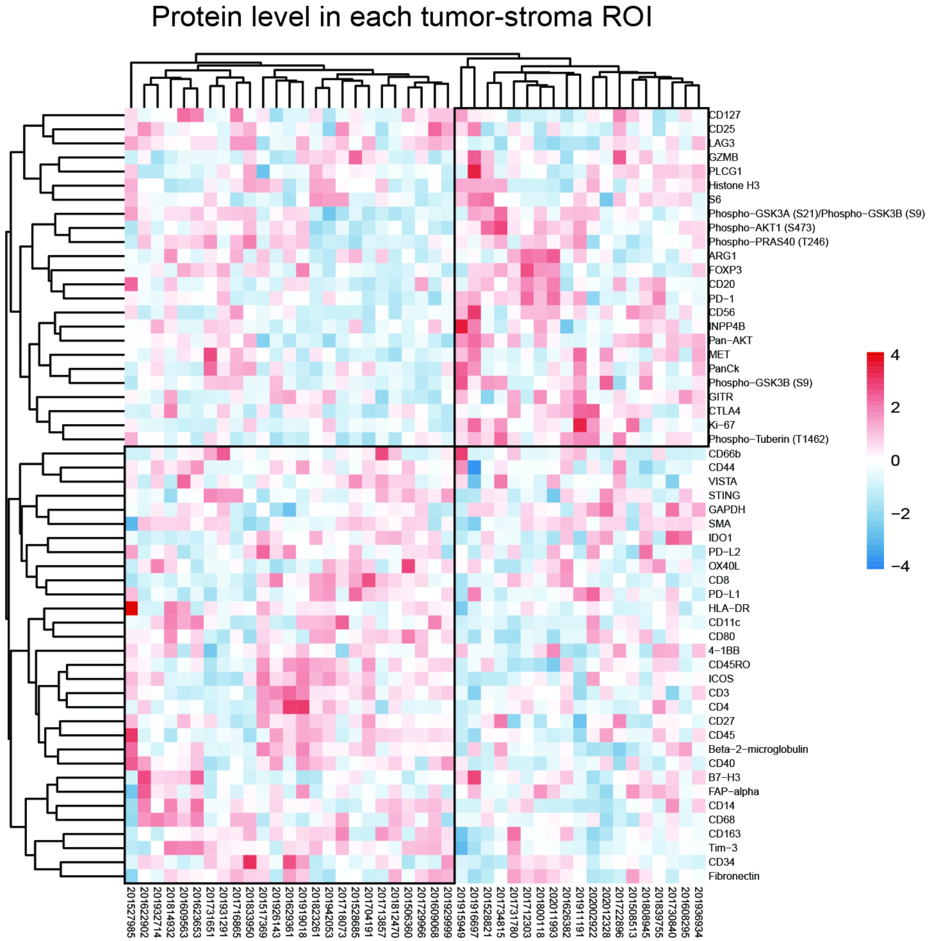

Supplementary Fig. S5

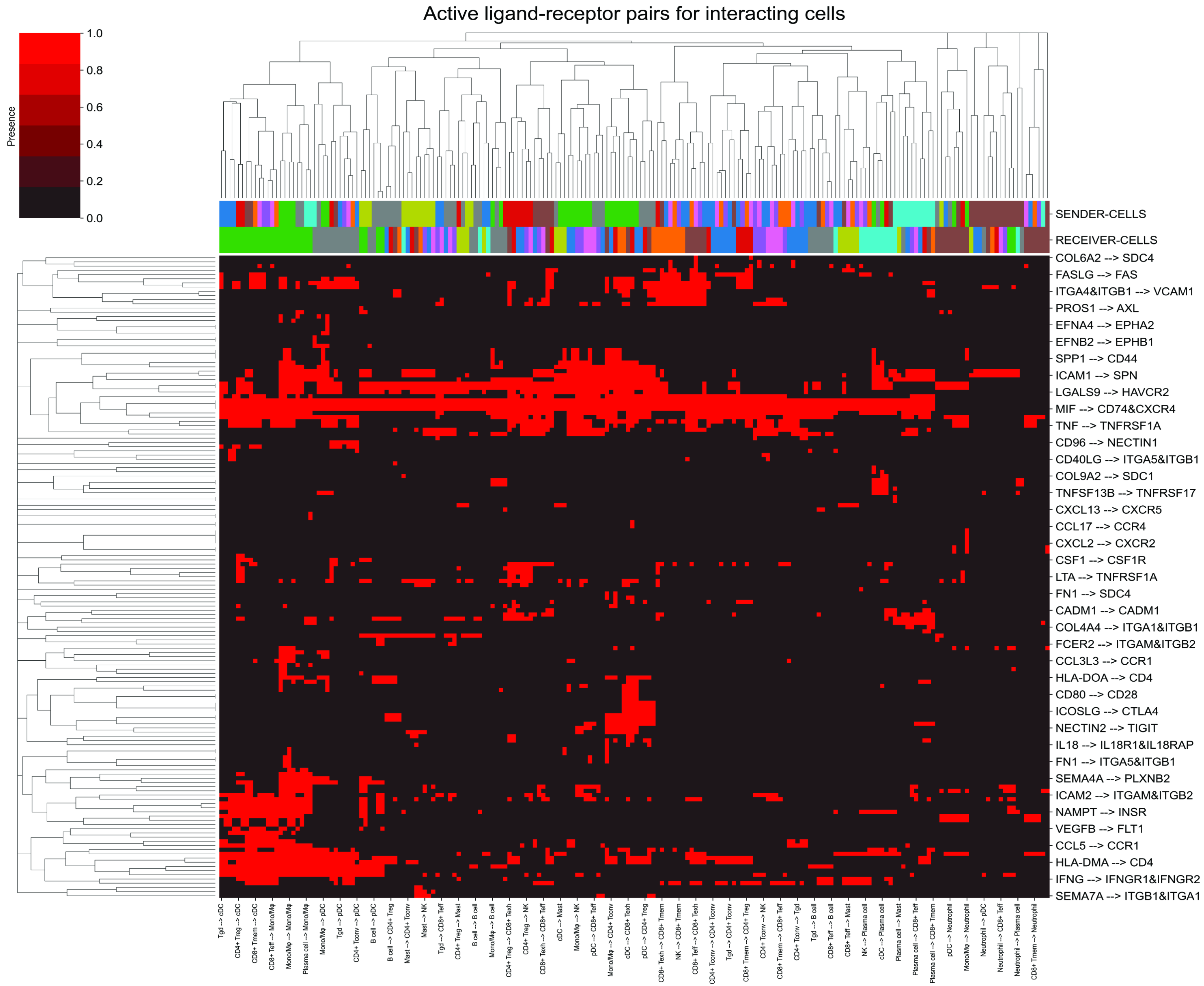

Supplementary Fig. S6

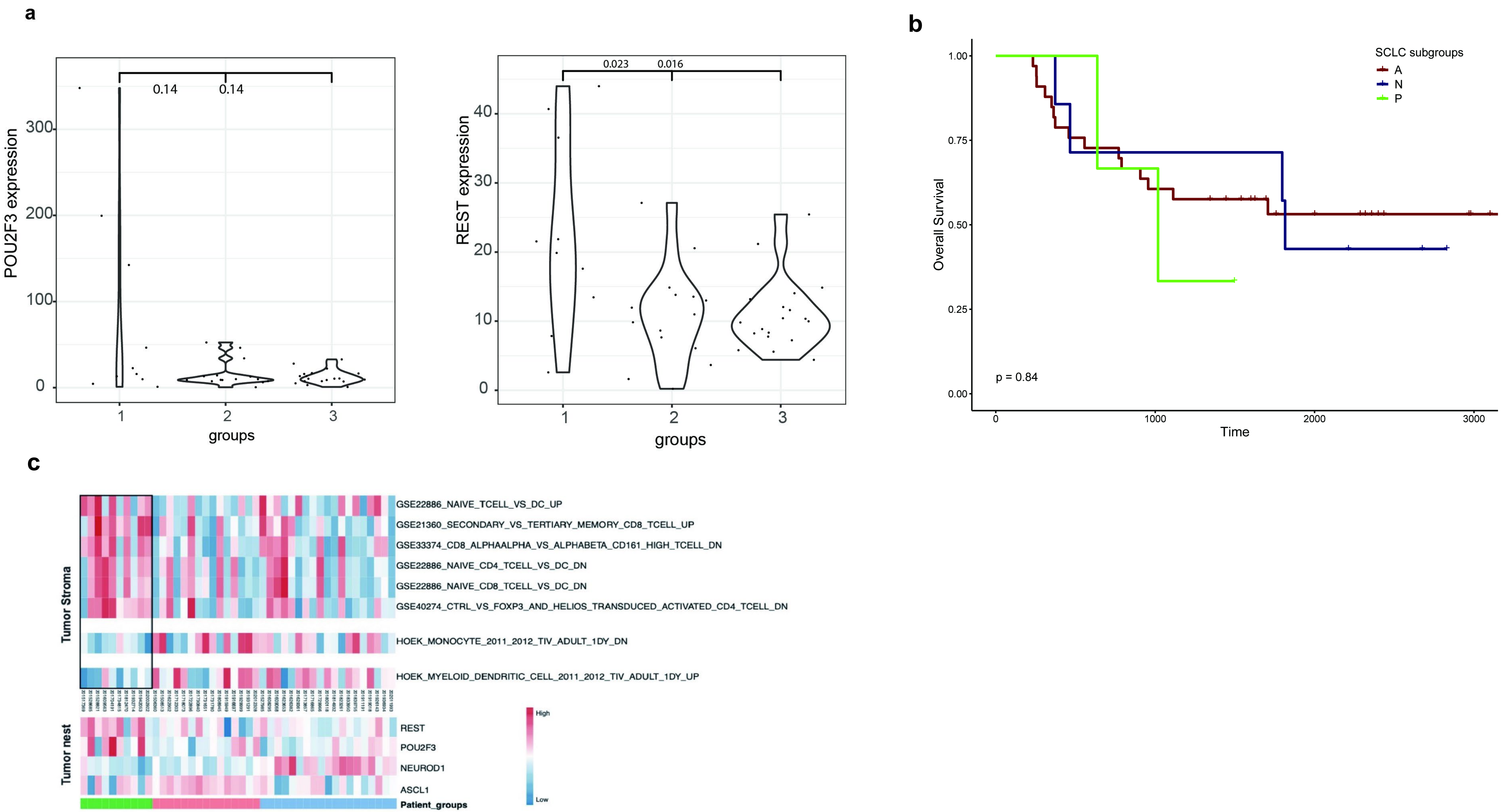

Supplementary Fig. S7

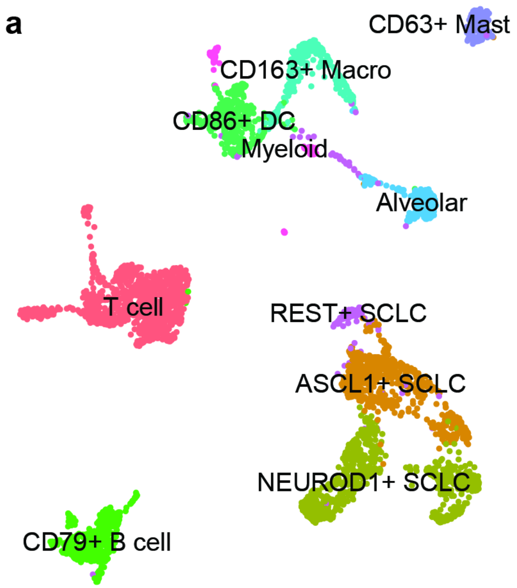

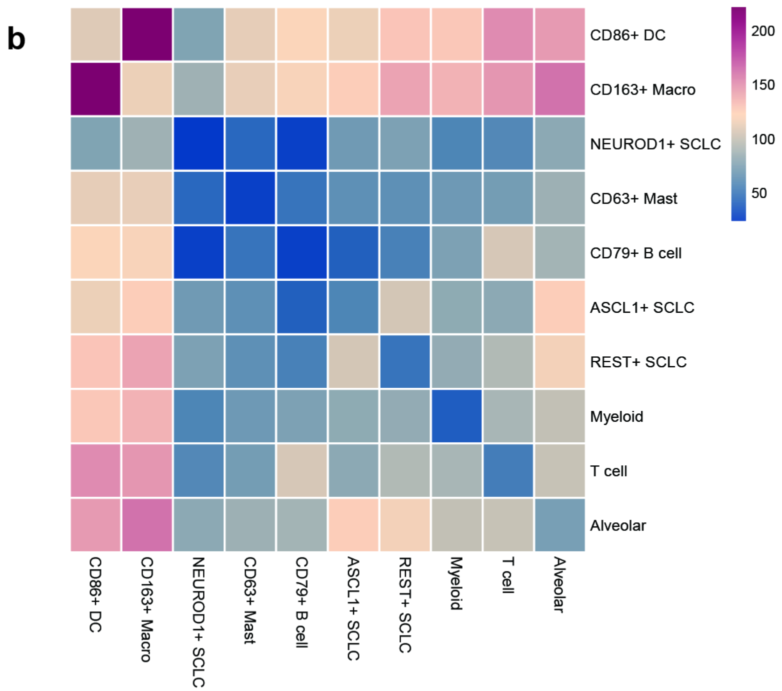

Supplementary Fig. S8

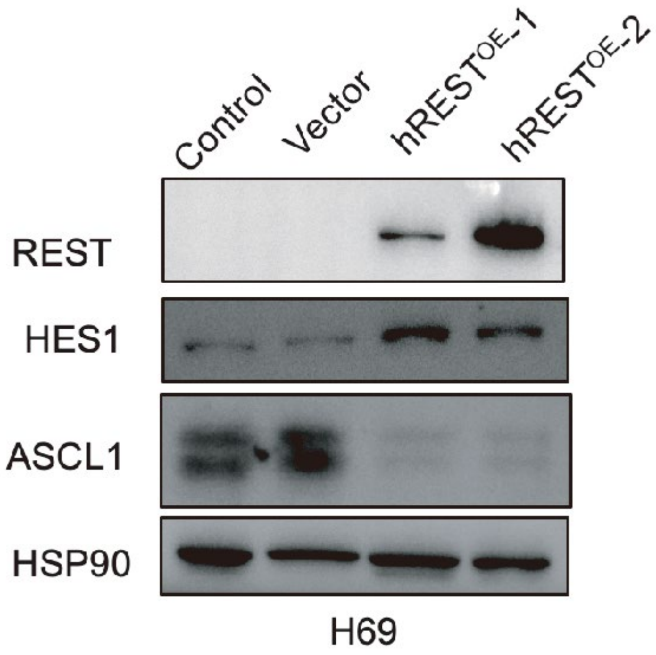

Supplementary Fig. S9

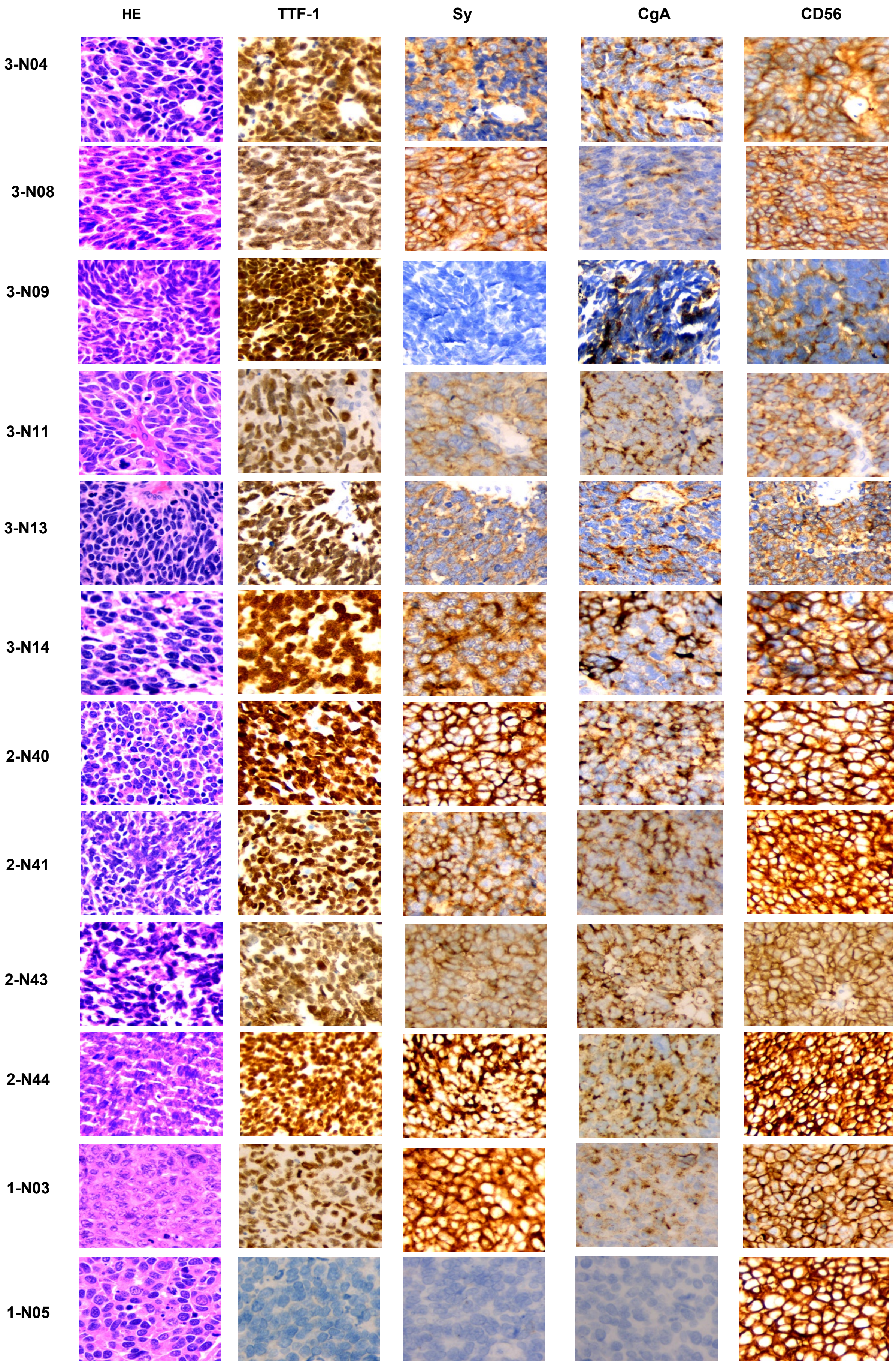

Supplementary Fig. S1. Single cell RNA-seq analysis from various tissues of SCLC and large cell neuroendocrine carcinomas. a) The abundance of major immune subsets in each sample source. **b)** DDRtree clustering of the cancer cells with the differentially expressed genes selected using un-supervised algorithm. **c)** The abundance of immune subsets in each sample.

Supplementary Fig. S2. Kaplan-Meier survival curves of 44 LS-SCLC patients based on the median protein level of a) CD4, b) CD3, c) CD8, d) PD-L1(TPS), e) PD-L1(CPS) by immunohistochemistry. f) TLS scores derived from WTA data in tumor nests versus tumor stroma ROIs.

Supplementary Fig. S3. Protein marker levels in tumor nest (green), tumor stoma (red) and para-tumor (blue) regions. Significance was tested using Wilcoxon test.

Supplementary Fig. S4. Protein marker levels in tumor-nest a) and tumor-stroma b) based on hierarchical clustering on both the protein markers and ROIs.

Supplementary Fig. S5 a) The communication scores for each ligand-receptor (LR) and cell pair. The lighter red color patches represent stronger interactions between sender and receiver cells.

Supplementary Fig. S6. a) The gene expression level of REST versus POU2F3 in the 3 NMF-defined groups (Wilcoxon test). **b)** Kaplan-Meier survival of 44 LS-SCLC patients based on previously defined molecular subtypes SCLC-A, N, P. **c)** The expression of association between patient tumor subtypes and corresponding tumor stroma immune cell abundance.

Supplementary Fig. S7. The validation of the REST+ SCLC cell existence and their interactions with immune cells. a) The UMAP plot of the cells and their annotations. **b)** The heatmap showing the interacted ligand-receptor pair numbers.

Supplementary Fig. S8. The western blotting quantification of ASCL1, HES1 and REST in H69 cell line with REST expression and control groups.

Supplementary Fig. S9. H&E and IHC images of 10 cases of SCLC and 2 cases of LCNEC.

Supplementary Table 1

Clinical and pathologic features for the 5 cases included for single cell sequencing.

ID	Age	Gender	Smoking	Date of biopsy/surgery	Samples for analysis	Pathologic diagnosis	Date of diagnosis	TNM stage	Treatment regimen	Best of response	Chest ratiotherapy
1-N01	69	male	Yes	2020/4/23	Pleural effusion, Lymph node, PBMC	SCLC	2020/4/28	cT4N3M1	EC (4 cycles)	SD	No
1-N02	55	male	Yes	2020/10/30	liver metastasis, PBMC	SCLC	2020/11/2	cT1N2M1	Tirelizumab+EC (6 cycles)	PR	No
1-N03	67	male	Yes	2020/11/10	Primary lesion, Para-tumor Control, Lymph node, PBMC	LCNEC	2020/11/20	pT1N2M0	Radical resection+EP (2 cycles)	NA	No
1-N04	65	male	Yes	2021/3/11	Primary lesion, Lymph node, PBMC	SCLC	2021/3/17	cT4N3M1	EC (1 cycles)	Unknown	No
1-N05	52	male	Yes	2021/5/18	Primary lesion, Para-tumor Control, PBMC	LCNEC	2021/5/22	pT3N1M0	Radical resection+EP (4 cycles)	NA	No

SCLC: small-cell lung cancer; LCNEC: large-cell neuroendocrine cancer; PBMC: peripheral blood mononuclear cell; EC: Etoposide+Carboplatin; EP:Etoposide+Cisplatin

Clinical and pathologic features for the 44 SCLCs included for DSP.

ID	Age	Gender	Smoking	Date of surgery	Samples for analysis	Pathologic diagnosis	Date of diagnosis	TNM stage	Adjuvant chemotherapy	PCI	Chest ratiotherapy
2-N01	56	male	Yes	2015/3/27	3 ROIs of Tumor, 3 ROIs of Stroma	SCLC	2015/4/7	pT1N0M0	EP (4 cycles)	No	No
2-N02	59	male	Yes	2015/4/21	3 ROIs of Tumor, 1 ROI of Stroma, 1 ROI of Para-tumor	SCLC	2015/4/27	pT1N0M0	EP (4 cycles)	No	No
2-N03	61	male	Yes	2015/7/27	2 ROIs of Tumor, 2 ROIs of Stroma, 1 ROI of Para-tumor	SCLC	2015/8/5	pT1N0M0	No	No	No
2-N04	48	female	No	2015/11/27	2 ROIs of Tumor, 1 ROI of Stroma, 1 ROI of Para-tumor	SCLC	2015/12/9	pT1N1M0	EP (4 cycles)	No	Yes
2-N05	72	male	Yes	2015/12/7	2 ROIs of Tumor, 2 ROIs of Stroma, 1 ROI of Para-tumor	SCLC	2015/12/16	pT1N0M0	EC (4 cycles)	No	No
2-N06	67	male	Yes	2015/12/8	2 ROIs of Tumor, 2 ROIs of Stroma, 1 ROI of Para-tumor	SCLC	2015/12/18	pT1N0M0	EC (6 cycles)	No	No
2-N07	50	male	Yes	2016/4/15	3 ROIs of Tumor, 2 ROIs of Stroma, 1 ROI of Para-tumor	SCLC	2016/4/26	pT2N1M0	No	No	No
2-N08	38	male	Yes	2016/4/25	2 ROIs of Tumor, 2 ROIs of Stroma, 1 ROI of Para-tumor	SCLC	2016/5/16	pT2N0M0	EP (4 cycles)	No	No
2-N09	62	male	Yes	2016/4/28	3 ROIs of Tumor, 2 ROIs of Stroma, 1 ROI of Para-tumor	SCLC	2016/5/9	pT2N0M0	EC (4 cycles)	No	No
2-N10	71	male	Yes	2016/9/22	2 ROIs of Tumor, 1 ROI of Stroma, 1 ROI of Para-tumor	SCLC	2016/10/25	pT3N0M0	EP (4 cycles)	No	No
2-N11	76	male	Yes	2016/9/29	3 ROIs of Tumor, 3 ROIs of Stroma, 1 ROI of Para-tumor	SCLC	2016/10/13	pT1N1M0	EP (4 cycles)	No	Yes
2-N12	61	male	Yes	2016/10/31	2 ROIs of Tumor, 2 ROIs of Stroma, 1 ROI of Para-tumor	SCLC	2016/11/8	pT1N0M0	EP (4 cycles)	No	No
2-N13	70	male	No	2016/11/28	3 ROIs of Tumor, 3 ROIs of Stroma, 1 ROI of Para-tumor	SCLC	2016/12/7	pT1N0M0	EP (1 cycle)	No	No
2-N14	68	male	Yes	2017/2/21	3 ROIs of Tumor, 3 ROIs of Stroma, 1 ROI of Para-tumor	SCLC	2017/3/2	pT2N0M0	EP (4 cycles)	No	No
2-N15	76	male	Yes	2017/5/8	3 ROIs of Tumor, 2 ROIs of Stroma, 1 ROI of Para-tumor	SCLC	2017/5/19	pT1N0M0	No	No	No
2-N16	72	male	No	2017/5/25	3 ROIs of Tumor, 2 ROIs of Stroma, 1 ROI of Para-tumor	SCLC	2017/6/6	pT1N0M0	EC (5 cycles)	No	No
2-N17	71	female	No	2017/6/19	2 ROIs of Tumor, 2 ROIs of Stroma, 1 ROI of Para-tumor	SCLC	2017/6/29	pT3N2M0	EP (2 cycles)	No	No
2-N18	63	male	Yes	2017/6/29	2 ROIs of Tumor, 2 ROIs of Stroma, 1 ROI of Para-tumor	SCLC	2017/7/10	pT1N1M0	EP (4 cycles)	Yes	Yes
2-N19	72	male	Yes	2017/8/10	2 ROIs of Tumor, 2 ROIs of Stroma, 1 ROI of Para-tumor	SCLC	2017/8/23	pT2N0M0	EC (4 cycles)	Yes	No
2-N20	71	male	No	2017/10/20	2 ROIs of Tumor, 2 ROIs of Stroma, 1 ROI of Para-tumor	SCLC	2017/10/30	pT1N2M0	EP (1 cycle)	No	No
2-N21	55	male	Yes	2017/10/27	2 ROIs of Tumor, 2 ROIs of Stroma, 1 ROI of Para-tumor	SCLC	2017/11/7	pT1N1M0	EP (4 cycles)	Yes	Yes
2-N22	65	male	Yes	2017/11/3	3 ROIs of Tumor, 2 ROIs of Stroma, 1 ROI of Para-tumor	SCLC	2017/11/16	pT1N1M0	EP (4 cycles)	Yes	Yes
2-N23	69	male	Yes	2017/11/6	3 ROIs of Tumor, 3 ROIs of Stroma, 1 ROI of Para-tumor	SCLC	2017/11/20	pT2N1M0	EP (4 cycles)； EC（2 cycles）	No	No
2-N24	68	male	Yes	2017/12/4	2 ROIs of Tumor, 2 ROIs of Stroma, 1 ROI of Para-tumor	SCLC	2017/12/13	pT2N0M0	EP (4 cycles)	No	No
2-N25	57	female	No	2018/1/2	3 ROIs of Tumor, 2 ROIs of Stroma, 1 ROI of Para-tumor	SCLC	2018/1/10	pT1N0M0	EP (4 cycles)； EC（2 cycles）	No	No
2-N26	51	male	Yes	2018/4/3	3 ROIs of Tumor, 2 ROIs of Stroma, 1 ROI of Para-tumor	SCLC	2018/4/12	pT1N0M0	EP (4 cycles)	No	No
2-N27	74	male	No	2018/5/7	3 ROIs of Tumor, 2 ROIs of Stroma, 1 ROI of Para-tumor	SCLC	2018/5/22	pT1N0M0	No	No	No
2-N28	55	male	Yes	2018/5/25	3 ROIs of Tumor, 3 ROIs of Stroma, 1 ROI of Para-tumor	SCLC	2018/6/5	pT2N1M0	EP (4 cycles)	Yes	Yes
2-N29	66	male	Yes	2018/8/2	3 ROIs of Tumor, 3 ROIs of Stroma, 1 ROI of Para-tumor	SCLC	2018/8/16	pT1N1M0	EP (4 cycles)	Yes	No
2-N30	74	male	Yes	2018/11/6	3 ROIs of Tumor, 2 ROIs of Stroma, 1 ROI of Para-tumor	SCLC	2018/11/15	pT3N0M0	EC (1 cycle)	No	No
2-N31	67	male	Yes	2018/12/24	2 ROIs of Tumor, 2 ROIs of Stroma, 1 ROI of Para-tumor	SCLC	2019/1/3	pT1N0M0	EP (2 cycles)	No	No
2-N32	70	male	Yes	2019/4/18	3 ROIs of Tumor, 2 ROIs of Stroma, 1 ROI of Para-tumor	SCLC	2019/4/30	pT2N0M0	EC (4 cycles)	No	No
2-N33	68	female	No	2019/5/28	2 ROIs of Tumor, 1 ROI of Stroma, 1 ROI of Para-tumor	SCLC	2019/5/31	pT1N1M0	No	No	No
2-N34	57	male	Yes	2019/6/3	1 ROI of Tumor, 1 ROI of Stroma, 1 ROI of Para-tumor	SCLC	2019/6/12	pT1N1M0	EP (4 cycles)	Yes	Yes
2-N35	59	male	Yes	2019/6/20	3 ROIs of Tumor, 3 ROIs of Stroma, 1 ROI of Para-tumor	SCLC	2019/6/26	pT1N1M0	EP (4 cycles)	Yes	Yes
2-N36	74	male	Yes	2019/8/12	2 ROIs of Tumor, 2 ROIs of Stroma, 1 ROI of Para-tumor	SCLC	2019/8/19	pT1N1M0	EC (4 cycles)	No	Yes
2-N37	64	male	Yes	2019/9/11	2 ROIs of Tumor, 2 ROIs of Stroma, 1 ROI of Para-tumor	SCLC	2019/9/19	pT1N1M0	EP (4 cycles)	No	No
2-N38	82	male	Yes	2019/9/23	3 ROIs of Tumor, 3 ROIs of Stroma, 1 ROI of Para-tumor	SCLC	2019/9/30	pT1N0M0	EC (4 cycles)	No	No
2-N39	66	male	Yes	2019/10/9	3 ROIs of Tumor, 3 ROIs of Stroma, 1 ROI of Para-tumor	SCLC	2019/10/15	pT1N1M0	EP (4 cycles)	No	Yes
2-N40	54	female	No	2019/11/7	3 ROIs of Tumor, 3 ROIs of Stroma, 1 ROI of Para-tumor	SCLC	2019/11/18	pT3N0M0	EP (1 cycle)	No	No
2-N41	75	male	Yes	2019/12/19	2 ROIs of Tumor, 2 ROIs of Stroma, 1 ROI of Para-tumor	SCLC	2019/12/26	pT1N1M0	EC (3 cycles)	Yes	Yes
2-N42	63	male	No	2020/2/10	2 ROIs of Tumor, 1 ROI of Stroma, 1 ROI of Para-tumor	SCLC	2020/2/17	pT1N0M0	EP (4 cycles)	No	Yes
2-N43	70	male	Yes	2020/5/18	3 ROIs of Tumor, 2 ROIs of Stroma, 1 ROI of Para-tumor	SCLC	2020/5/25	pT2N2M0	EP (3 cycles)； EC（1 cycle）	Yes	Yes
2-N44	65	male	Yes	2020/5/20	2 ROIs of Tumor, 1 ROI of Stroma, 1 ROI of Para-tumor	SCLC	2020/5/27	pT1N1M0	EP (4 cycles)	Yes	Yes

SCLC: small-cell lung cancer; ROI:Region of interest; EC: Etoposide+Carboplatin; EP:Etoposide+Cisplatin； PCI：Prophylactic cranial irradiation

Clinical and pathologic features for the 16 SCLCs （16 tumors; 4 para-tumors） included for single cell sequencing.

ID	Age	Gender	Smoking	Date of surgery	Samples for analysis	Pathologic diagnosis	Date of diagnosis	TNM stage	Adjuvant chemotherapy	PCI	Chest ratiotherapy
3-N01	67	male	Yes	2022/2/22	Tumor (primary lesion)	SCLC	2022/2/28	pT1N1M0	EC (3 cycles)	Yes	No
3-N02	66	male	No	2022/3/30	Tumor (primary lesion)	SCLC	2022/4/6	pT1N0M0	No	No	No
3-N03	59	male	No	2022/5/24	Tumor (primary lesion)	SCLC	2022/5/30	pT1N0M0	EC (4 cycles)	No	No
3-N04	60	male	Yes	2022/8/10	Tumor (primary lesion)	SCLC	2022/8/17	pT2N2M0	EP (4 cycles)	Yes	Yes
3-N05	73	male	No	2022/8/18	Tumor (primary lesion)	SCLC	2022/8/25	pT1N0M0	EC (2 cycles)	No	No
3-N06	69	male	No	2022/9/7	Tumor (primary lesion)	SCLC	2022/9/16	pT1N2M0	EC (4 cycles)	Yes	Yes
3-N07	66	male	Yes	2022/9/28	Tumor (primary lesion)	SCLC	2022/10/13	pT1N0M0	No	No	No
3-N08	71	male	Yes	2023/1/12	Tumor (primary lesion)	SCLC	2023/1/18	pT1N1M0	EC (4 cycles)	Yes	Yes
3-N09	61	male	Yes	2023/2/16	Tumor (primary lesion); Para-tumor Control	SCLC	2023/2/24	pT1N0M0	EC (4 cycles)	No	No
3-N10	68	male	No	2023/2/21	Tumor (primary lesion)	SCLC	2023/3/2	pT2N0M0	EC (4 cycles)	Yes	No
3-N11	70	male	No	2023/2/22	Tumor (primary lesion); Para-tumor Control	SCLC	2023/3/1	pT2N1M0	EC (4 cycles)	Yes	No
3-N12	70	male	No	2023/6/6	Tumor (primary lesion)	SCLC	2023/6/26	pT1N0M0	EC (4 cycles)	No	No
3-N13	69	female	No	2023/6/9	Tumor (primary lesion); Para-tumor Control	SCLC	2023/6/14	pT1N0M0	No	No	No
3-N14	69	male	No	2023/7/10	Tumor (primary lesion)	SCLC	2023/7/21	pT1N0M0	EC (4 cycles)	No	No
3-N15	76	male	Yes	2023/10/27	Tumor (primary lesion); Para-tumor Control	SCLC	2023/11/7	pT1N0M0	No	No	No
3-N16	68	male	Yes	2023/11/6	Tumor (primary lesion)	SCLC	2023/11/15	pT1N0M0	EC (4 cycles)	No	No

SCLC: small-cell lung cancer; EC: Etoposide+Carboplatin; EP:Etoposide+Cisplatin； PCI：Prophylactic cranial irradiation
